# Intermittent exposure to whole cigarette smoke alters the differentiation of primary small airway epithelial cells in the air-liquid interface culture

**DOI:** 10.1038/s41598-020-63345-5

**Published:** 2020-04-10

**Authors:** Julia A. Gindele, Tobias Kiechle, Kerstin Benediktus, Gerald Birk, Michael Brendel, Fabian Heinemann, Christian T. Wohnhaas, Michelle LeBlanc, Haijun Zhang, Yael Strulovici-Barel, Ronald G. Crystal, Matthew J. Thomas, Birgit Stierstorfer, Karsten Quast, Jürgen Schymeinsky

**Affiliations:** 1 Immunology & Respiratory Diseases Research, Boehringer Ingelheim Pharma GmbH & Co. KG, Biberach an der Riß, Germany; 2 Department of General Physiology, University of Ulm, Ulm, Germany; 3 Drug Discovery Sciences, Boehringer Ingelheim Pharma GmbH & Co. KG, Biberach an der Riß, Germany; 4 Non-Clinical Statistics Biberach, Boehringer Ingelheim Pharma GmbH & Co. KG, Biberach an der Riß, Germany; 5 Global Computational Biology and Digital Sciences, Boehringer Ingelheim Pharma GmbH & Co. KG, Biberach an der Riß, Germany; 6 Department of Biology, University of Konstanz, Konstanz, Germany; 7 Department of Genetic Medicine, Weill Cornell Medical College, New York, USA

**Keywords:** Respiratory system models, Mechanisms of disease, Chronic obstructive pulmonary disease

## Abstract

Cigarette smoke (CS) is the leading risk factor to develop COPD. Therefore, the pathologic effects of whole CS on the differentiation of primary small airway epithelial cells (SAEC) were investigated, using cells from three healthy donors and three COPD patients, cultured under ALI (air-liquid interface) conditions. The analysis of the epithelial physiology demonstrated that CS impaired barrier formation and reduced cilia beat activity. Although, COPD-derived ALI cultures preserved some features known from COPD patients, CS-induced effects were similarly pronounced in ALI cultures from patients compared to healthy controls. RNA sequencing analyses revealed the deregulation of marker genes for basal and secretory cells upon CS exposure. The comparison between gene signatures obtained from the *in vitro* model (CS vs. air) with a published data set from human epithelial brushes (smoker vs. non-smoker) revealed a high degree of similarity between deregulated genes and pathways induced by CS. Taken together, whole cigarette smoke alters the differentiation of small airway basal cells *in vitro*. The established model showed a good translatability to the situation *in vivo*. Thus, the model can help to identify and test novel therapeutic approaches to restore the impaired epithelial repair mechanisms in COPD, which is still a high medical need.

## Introduction

Chronic obstructive pulmonary disease (COPD) is the third leading cause of death worldwide and its prevalence continues to rise^1^. The main risk factor to develop COPD is cigarette smoke^2,3^. Smoking induces epithelial injury and this repeated injury of the epithelium triggers a pathophysiologic response, which leads to tissue remodeling of the airways that is characteristic for COPD^4,5^. These changes of the small airway epithelium in COPD include: Goblet cell metaplasia^6–8^, reduced cilia function^9–13^, reduced club cell numbers^7,14,15^, basal membrane thickening^16,17^, epithelial barrier dysfunction^18–20^ and squamous metaplasia^8,21–23^. Furthermore, the epithelial defense mechanisms against inhaled particles and pathogens are impaired enabling sub-epithelial penetration of pathogens that increases the risk of COPD patients suffering from bacterial and viral infections and subsequent exacerbations^24–26^.

To address cigarette smoke (CS)-induced damage on epithelial cells *in vitro*, previous studies used primary epithelial cells or cell lines that were mostly exposed to cigarette smoke extract (CSE) or to whole CS. These studies demonstrate smoke effects e.g. on epithelial barrier integrity, mucus production and cilia toxicity^27–34^. The majority of these studies focus on the pathophysiology of large airways, i.e. bronchial or tracheal epithelial cells.

One hallmark of COPD is chronic bronchiolitis that mainly affects the small airways^17^. Therefore, a novel protocol was developed to culture human small airway epithelial cells (SAEC) at the air-liquid interface (ALI). To model CS induced alterations of the small airway epithelium, SAEC were repeatedly exposed to whole cigarette smoke during the differentiation process. In order to address the question if smoking and health status has an impact on the sensitivity to cigarette smoke exposure, cells from three healthy non-smokers and three COPD-smokers were compared.

To assess, how the *in vitro* model translates to the situation *in vivo*, the gene expression profiles of the ALI cultures were compared to the profiles of human small airway samples from healthy non-smokers and healthy smokers.

## Results

### Establishment of SAEC ALI culture

**Figure 1:**
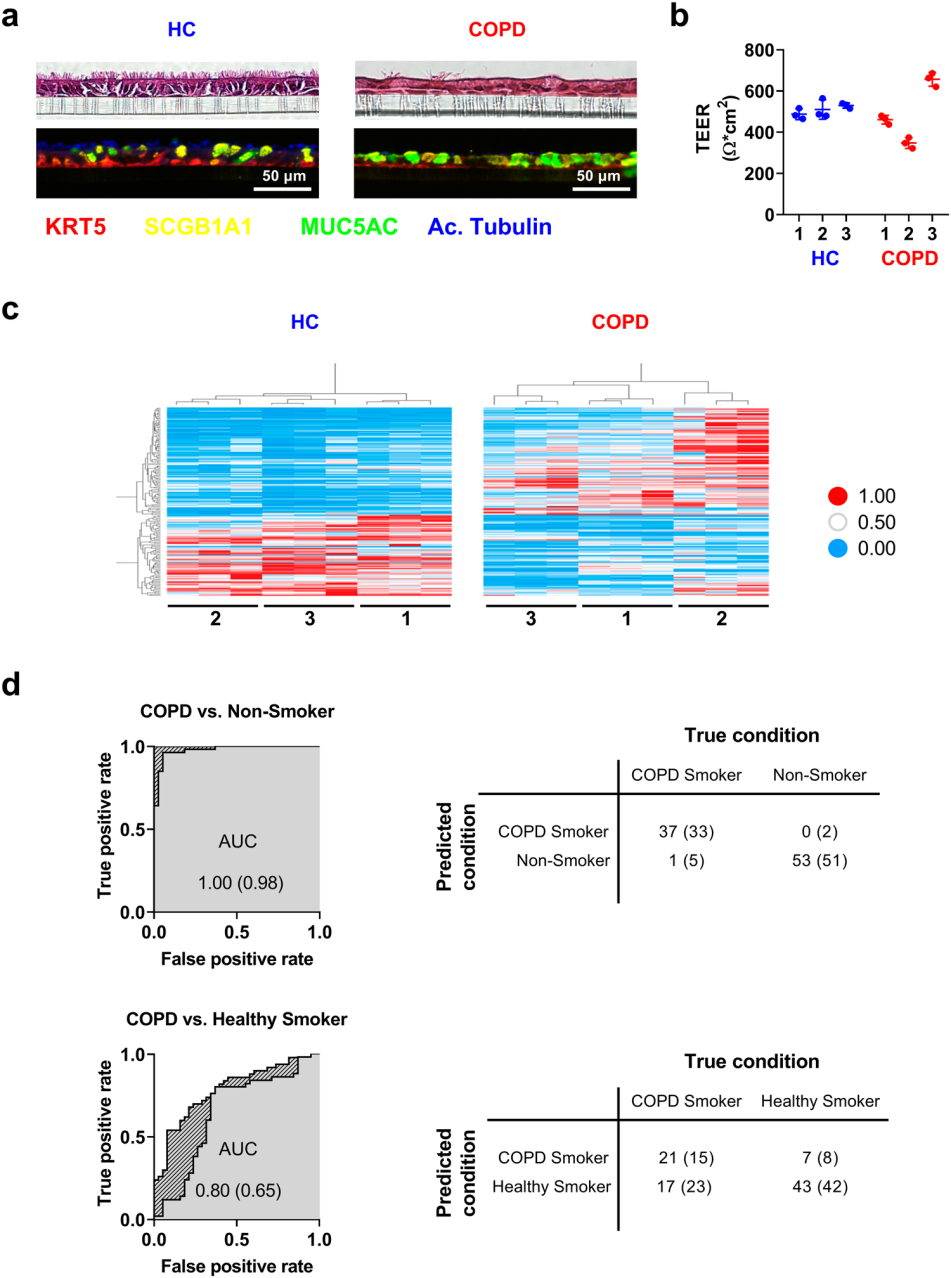
Comparison of SAEC culture obtained from donors with COPD and healthy controls. Fully differentiated SAEC from healthy controls (HC) and COPD donors were grown and analyzed at the air-liquid interface (ALI) for 28 days. (**a**) H&E and IHC stainings of sections of ALI cultures show pseudostratified epithelium with basal (KRT5), secretory (SCGB1A1, MUC5AC) and ciliated (acetylated tubulin) cells. (**b**) SAEC ALI cultures develop an intact epithelial barrier quantified by trans-epithelial electric resistance (TEER) measurements. (**c**) Heatmap representation of unbiased cluster analysis of 170 transcripts exhibiting a q Value <0.05 in comparing HC versus COPD. Absolute expression values were normalized to a range from 0 to 1 of SAEC ALI cultures. Numbers represent samples from same subjects (donor 1–3 HC and COPD, respectively). (**d**) ROC (receiver operating characteristic) curves and confusion matrices to characterize the classification power of the *in vitro* ALI COPD signature (predicted condition) towards a published data set from epithelial brushes of patients with COPD and healthy controls (true condition). A random forest classifier with 50-fold cross validation was used to classify COPD (n = 38) vs. non-smoker (n = 53) and COPD (n = 38) vs. healthy smoker (n = 50). SAECs from an independent cohort utilizing the ALI COPD signature as feature vector. Dashed regions indicate AUCs observed for the full signature, grey regions AUCs upon removal of the ten highest correlating transcripts. Numbers in parentheses represent values obtained for the reduced signature.

To further understand the molecular mechanisms underlying small airway disease and airway remodeling in COPD a new *in vitro* protocol was established for the cultivation of primary SAEC at ALI. First, the differentiation of SAEC under ALI conditions was characterized by analyzing the histology, the cellular composition (expression of cellular markers by RT-PCR technique), and the epithelial physiology (measuring transepithelial resistance and cilia beating). Removal of the apical medium (initiation of ALI culture) induced the differentiation of SAEC into a pseudostratified two-layered epithelium in 28 days (Fig. 1a). By means of immunohistochemistry, the main small airway cell types: KRT5^+^ basal cells, FOXJ1^+^ ciliated cells and SCGB1A1^+^ club cells were detected (Fig. 1a). MUC5AC^+^ goblet cells are normally absent in the human small airways^35^, however an intermediate cell population of SCGB1A1^+^/MUC5AC^+^ double positive secretory cells and a few SCGB1A1^−^/MUC5AC^+^ goblet cells were detected in the ALI cultures (Fig. 1a). For the characterization of the epithelial physiology, transepithelial electrical resistance (TEER; Fig. 1b) was measured and cilia beating was investigated using a high-speed camera, enabling the quantification of the area of ciliated cells and the frequency of cilia beating (Fig. S1). At day 28 post airlift, the ALI cultures established from SAECs have a typical TEER between 400–600 Ω*cm^2^ (Fig. 1b), a cilia beat frequency between 7–10 Hz and show an area of 5–20% covered by actively beating cilia.

### A COPD-like phenotype is conserved in ALI cultures derived from donors with COPD

SAECs from three different COPD donors and three healthy controls were used to test whether pathologic changes in the epithelial histology are preserved in the differentiated ALI cultures. Of note, histological slices from ALI cultures obtained from COPD patients show a reduced number of ciliated cells and an increased number of secretory cells in comparison to control ALI cultures from healthy donors (Fig. 1a). To characterize the differential mRNA expression in detail, a Next Generation Sequencing (NGS) analysis was performed of ALI cultures obtained from healthy donors and COPD patients at day 28 upon air-lift. 248 transcripts were detected that were significantly changed in COPD-derived cells compared to healthy volunteer-derived cells. Hierarchical clustering of these transcripts reveals different mRNA signatures in healthy and COPD cultures (Fig. 1c). To assess the ability of the COPD ALI signature to distinguish between human small airway epithelial (SAE) samples of healthy non-smokers and COPD smokers as well as between healthy smokers and COPD smokers, the signature was used as feature vector in a random forest classification analysis^36^. The power of the signature to separate the respective samples was tested by determining the ROC (receiver operating characteristic) curves  (Fig. 1d). Classifying samples from healthy non-smokers and COPD smokers resulted in an AUC of 1.0, indicating a nearly perfect separation of SAE samples by utilizing the ALI culture derived signature. To ensure, that the high classification power is not driven solely by very few well separating transcripts, but rather by a large proportion of the signature, the best correlating transcripts were identified. Subsequently, ten transcripts were removed from the signature, which showed the highest correlation. After reiterating the classification, an AUC of 0.98 was obtained. This indicates a clear relationship between the transcriptional changes within the ALI culture and the SAE samples, which is not dependent upon the top ten classifying transcripts. The high overlap between the *in vitro* derived signature and the clinical situation allows a good prediction of the health status. In the task of separating smokers from COPD smokers an AUC of 0.8 was obtained. Upon removal of the ten highest correlating transcripts, this drops to 0.65. Even though the classification power is lower in this case, it still indicates an overlap of COPD driven transcriptional changes in the ALI samples with the changes observed in SAE from human subjects.

### Cigarette smoke exposure impairs epithelial barrier integrity

To investigate the harmful effects of CS on SAEC differentiation and physiology the ALI cultures were intermittently exposed to well-defined doses of whole CS during the differentiation period of 28 days. Unlike commonly used exposure models that use cigarette smoke extract (CSE) in which many volatile components are lost during the extraction process, using whole CS exposes the cells to all volatile toxins contained within CS. Furthermore, this is likely to focus CS exposure to the apical cell layer and reduce exposure to the basal cell layer, in contrast to a stimulation with CSE added to the basal medium. It is assumed that this setting is an optimal framework to mimic the direct toxic effects of smoking on the small airway epithelium.

**Figure 2:**
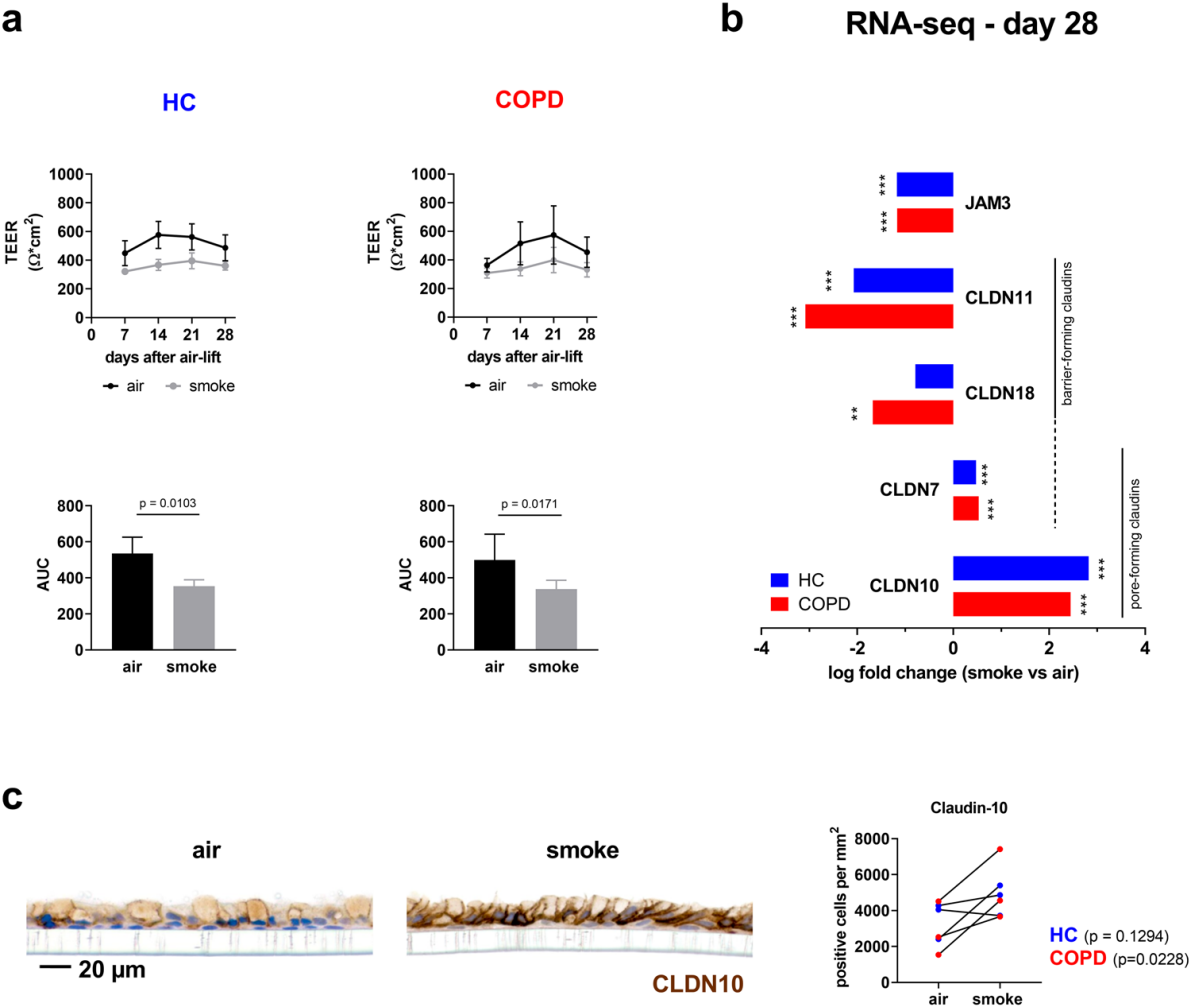
Cigarette smoke exposure impairs epithelial barrier integrity in ALI cultures from donors with COPD and healthy controls. (**a**) Upper panel: TEER measurements at days 7, 14, 21 and 28 from ALI cultures upon airlift. ALI cultures from patients with COPD and healthy controls (HC) were intermittently exposed with CS (grey line) or air for control (black line). n = 3 HC and 3 donors with COPD. Lower panel: Calculated area under the curve (AUC) to compare the TEER development over the observed time. Data shown as mean ± SEM; p values are based on the analysis of the log AUC values with a repeated measurement model. (**b**) Log fold changes of mRNA expression of junctional genes in ALI cultures induced by CS compared to air control at day 28 upon airlift. The exposure with CS led to a significant change of mRNA expression of genes related to various aspects of epithelial barrier integrity: *JAM3*, *CLDN11*, *CLDN18*, *CLDN7* and *CLDN10*. Data obtained from NGS analysis. Significance of deregulation according to a linear model applied to the normalized data is indicated as * for q value <0.05; ** for q value <0.01; *** for q value <0.001. n = 3 HC and 3 donors with COPD. (**c**) Representative images of an immunohistochemical staining for Claudin-10 (CLDN10) from a healthy SAEC ALI culture section at day 28 after air-lift with intermittent CS exposure (smoke) and air for control and semi-quantification using automated image analysis.

In order to assess effects of CS on epithelial barrier integrity, TEER was repeatedly measured in the ALI cultures during differentiation. Air control treated ALI cultures of healthy donors and COPD patients exhibited similar values at days 7, 14, 21 and 28 (Fig. 2a). However, the TEER values of CS exposed cultures were reduced compared to air controls (Fig. 2a). To quantify this effect the area under the curve (AUC) was calculated and standardized with respect to the time span. The adjusted AUC dropped in the healthy ALI cultures from 535 ± 91 Ω*cm^2^ in air controls to 354 ± 36 Ω*cm^2^ (p = 0.01) upon CS exposure, and in the COPD ALI cultures from 500 ± 143 Ω*cm^2^ to 337 ± 50 Ω*cm^2^ (p = 0,017), respectively (Fig. 2a). To investigate the underlying molecular mechanisms mRNA was extracted from the ALI cultures and the expression of junctional protein transcripts was analyzed. Some genes associated with cell adhesion, permeability and tight junction regulation were deregulated by CS. *JAM3*, *CLDN11* and *CLDN18* were down-regulated after 28 days of intermittent CS treatment. *CLDN7* and *CLDN10* were up-regulated in both healthy and COPD cultures (Fig. 2b). The upregulation of the strongest deregulated junctional protein on mRNA level, namely Claudin-10, was confirmed on protein level by means of immunohistochemistry (Fig. 2c). Of note, most of the prevalent classic claudins in the lung^37^, i.e. claudin-1, -3, -4, and -5, were not significantly deregulated on mRNA level (data not shown).

### Cigarette smoke exposure induces squamous differentiation

**Figure 3:**
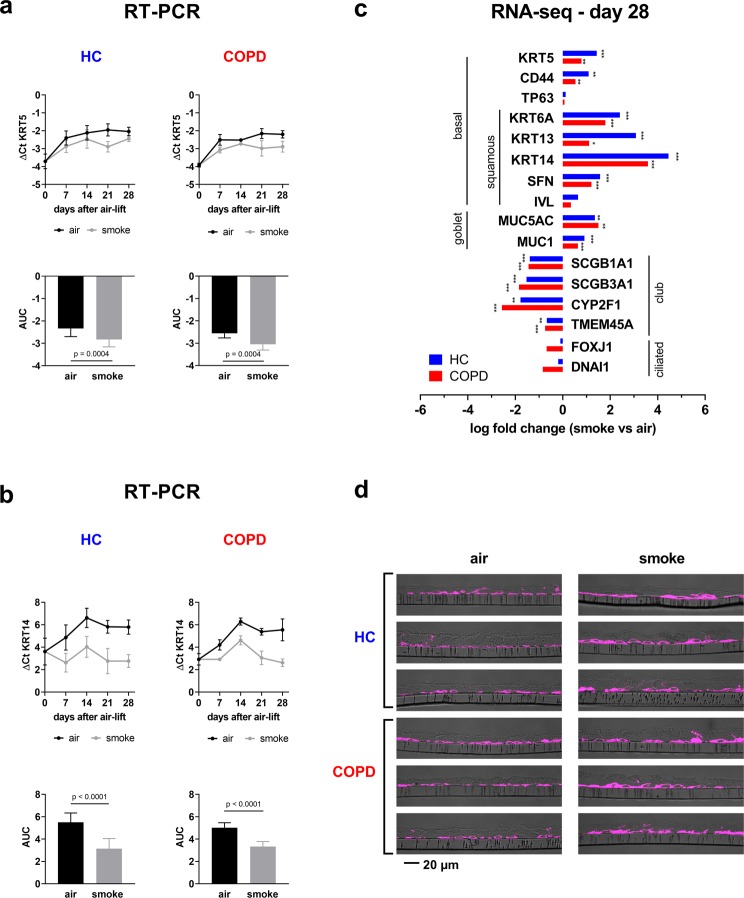
Cigarette smoke exposure alters the expression of gene markers for basal cells and induces squamous differentiation. Quantitative RT-PCR analysis of (**a**) the basal cell marker *KRT5* and (**b**) the squamous cell marker *KRT14*. The data were generated at day 7, 14, 21 and 28 upon air-lift from ALI cultures with intermittent CS exposure and air for control from donors with COPD (n = 3) and healthy controls (HC; n = 3). Adjusted area under the curve (AUC) was calculated to compare the mRNA development over the time. Data show mean ± SEM, p values are based on the analysis of the AUC values with a repeated measurement model. (**c**) RNA sequencing analysis (NGS) revealed log fold changes of mRNA expression of epithelial marker genes in ALI cultures induced by CS compared to air control at day 28 upon airlift. The exposure with CS led to a significant change of mRNA expression of genes related to aspects of airway epithelial differentiation: *KRT5*, *CD44*, *TP63*, *KRT6A*, *KRT13*, *KRT14*, *SFN*, *IVL*, *MUC5AC*, *MUC1*, *SCGB1A1*, *SCGB3A1*, *CYP2F1*, *TMEM45A*, *FOXJ1* and *DNAI1*. Data obtained from NGS analysis. Significance of deregulation according to a linear model applied to the normalized data is indicated as * for q value <0.05; ** for q value <0.01; *** for q value <0.001. n = 3 HC and 3 donors with COPD. (**d**) Immunohistochemical staining of ALI sections of day 28 after air-lift with intermittent CS exposure (smoke) and air for control, stained for KRT5. Representative images of cultures from three healthy donors and three COPD patients are shown.

One hallmark of COPD is squamous metaplasia^21^. Therefore, the expression of different squamous cell marker genes was investigated during the differentiation of smoked and control ALI cultures (RNA-seq, Fig. 3c). During the differentiation of the ALI cultures, the difference of threshold cycle values (∆Ct: Ct target gene – Ct endogenous control) for Keratin 5 (*KRT5*) increased from the day of airlift until day 14, where it reached a plateau, indicating that *KRT5* mRNA expression is downregulated during the normal differentiation process. This was true for ALI cultures from healthy and COPD SAECs. However, the exposure to CS led to a decrease of the ∆Ct values which reflects an increase of *KRT5* mRNA expression in healthy as well as in COPD ALI cultures (Fig. 3a). To quantify this effect over the observed time the AUC was calculated and standardized with respect to the time span. The exposure of ALI cultures to CS led to a decreased AUC which was similar in healthy (from −2.3 ± 0.4 ∆Ct to −2.8 ± 0.3 ∆Ct, p = 0.0004) and COPD derived cultures (from −2.6 ± 0.1 ∆Ct to −3.0 ± 0.1 ∆Ct, p = 0.0004) (Fig. 3b). The ∆Ct values for the squamous differentiation marker KRT14 increased during the ALI differentiation indicating that *KRT14* mRNA expression is downregulated during the normal differentiation process. However, the ∆Ct values were not down regulated during the differentiation of CS exposed cultures (Fig. 3b) indicating that CS-exposure is blocking the physiologic downregulation of *KRT14* expression. Accordingly, the AUC was downregulated upon CS exposure in ALI cultures from COPD patients (from 5.0 ± 0.3 ∆Ct to 3.3 ± 0.3 ∆Ct, p < 0.0001) and healthy controls (from 5.5 ± 0.8 ∆Ct to 3.1 ± 0.9 ∆Ct, p < 0.0001) (Fig. 3b). RNA sequencing analysis revealed deregulation of several cell marker genes, i.e. genes associated with basal cells, goblet and club cells as well as ciliated cells (Fig. 3c). *KRT5* and *KRT14* up-regulation was confirmed and the expression of other genes associated with squamous differentiation, namely Keratin 6 A (*KRT6A*), Keratin 13 (*KRT13*), and Stratifin (*SFN*) were found to be also up-regulated upon CS exposure, indicated by a positive fold-change (Fig. 3c). Besides evaluation on RNA level, KRT5 expression was assessed on protein level, qualitatively and representatively (Fig. 3d). Deregulation of secretory and ciliated markers were further evaluated in the following sections.

### Cigarette smoke exposure stimulates the differentiation of *MUC5AC*^+^ cells and reduces *SCGB1A1*^+^*cells*

**Figure 4:**
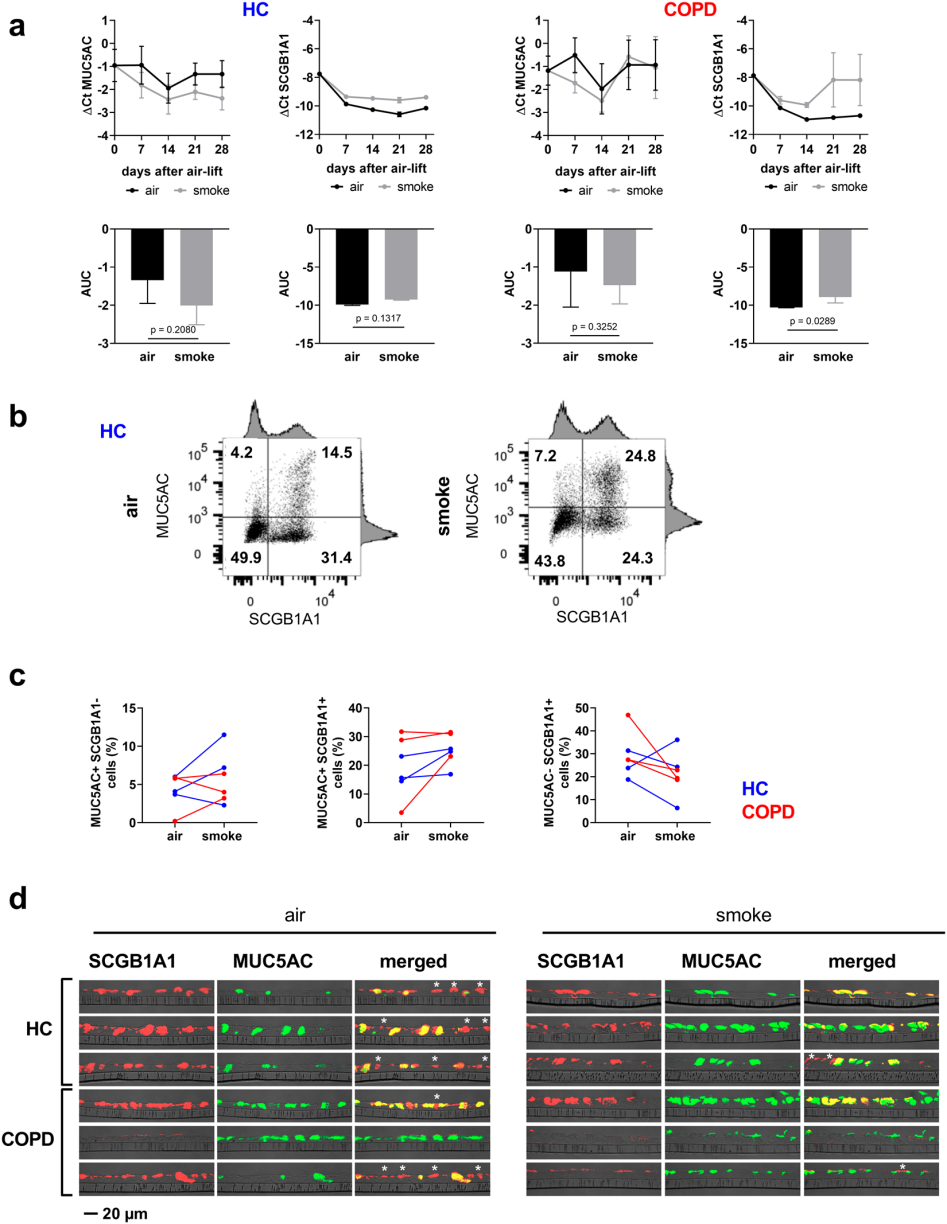
Cigarette smoke exposure stimulates the differentiation of MUC5AC^+^ goblet cells and reduces SCGB1A1^+^ club cells. (**a**) Upper panel: Quantitative RT-PCR analysis of the goblet cell marker *MUC5AC* and the club cell marker *SCGB1A1*. The data were generated at day 7, 14, 21 and 28 of ALI culture upon air-lift. The cultures were treated with intermittent exposure of CS or air for control. n = 3 donors with COPD and 3 healthy controls (HC). Lower panel: Adjusted area under the curve (AUC) was calculated to compare the mRNA development over the time. Data show mean ± SEM, p values are based on the analysis of the AUC values with a repeated measurement model. (**b**) Flow cytometry analysis of MUC5AC^+^/SCGB1A1^−^ single positive, MUC5AC^+^/SCGB1A1^+^ double positive and MUC5AC^−^/SCGB1A1^+^ single positive cells on day 28 upon air-lift. The scatter plot of one representative experiment is shown. (**c**) Quantitative analysis of the FACS analyses of 3 donors with COPD (red) and 3 healthy controls (blue). Data are shown as mean ± SEM. (**d**) Immunohistochemical staining of ALI sections of day 28 after air-lift with intermittent CS exposure (smoke) and air for control, stained for MUC5AC and SCGB1A1. SCGB1A1 single positive cells are marked by an asterix in the merged images. Representative images of cultures from three healthy donors and three COPD patients are shown.

To investigate the effect of CS on basal cell differentiation into secretory cells, the club cell marker *SCGB1A1* and the goblet cell marker *MUC5AC* were analyzed on mRNA level at day 7, 14, 21 and 28 after air-lift. The ΔCt values decreased after the day of air-lift, indicating an induction of *MUC5AC* and *SCGB1A1* mRNA expression, which goes along with the normal differentiation process. The CS exposed cultures exhibit lower ΔCt values for *MUC5AC*, indicating an increased *MUC5AC* mRNA expression. At the same time, ΔCt values for *SCGB1A1* were increased during differentiation, indicating a reduced *SCGB1A1* mRNA expression (Fig. 4a). The adjusted AUC was calculated to compare the differentiation into secretory cells of smoke exposed cultures and air controls. The AUC for MUC5AC was down-regulated upon CS exposure in ALI cultures from COPD patients (from −1.1 ± 0.9 ∆Ct to –1.5 ± 0.5 ∆Ct, p = 0.3252) and healthy controls (from −1.3 ± 0.6 ∆Ct to −2.0 ± 0.5 ∆Ct, p = 0.2080). This indicates an approximate increase of MUC5AC mRNA expression of 1.3 fold in the COPD cultures and 1.6 fold in the healthy ALI cultures. The AUC for SCGB1A1 was up-regulated in CS-exposed ALI cultures from COPD patients (from −10.3 ± 0.03 ∆Ct to −8.9 ± 0.8 ∆Ct, p = 0.0289) and healthy controls (from −9.9 ± 0.1 ∆Ct to −9.3 ± 0.1 ∆Ct, p = 0.1317). This indicates an approximate reduction of SCGB1A1 mRNA expression of 2.6 fold in the COPD cultures and 1.6 fold in the healthy ALI cultures. Furthermore, a RNA sequencing analysis indicated an increase of goblet cell associated genes (*MUC5AC*, *MUC1*) and a reduction of club cell genes (*SCGB1A1*, *SCGB3A1*, *CYP2F1*, *TMEM45A*) upon CS treatment (Fig. 3c).

To investigate if the changes on mRNA level led to altered protein expression and cell composition MUC5AC and SCGB1A1 protein expression were analyzed by means of FACS analysis. The analysis revealed for the majority of donors (five out of six) a decrease in SCGB1A1^+^ single positive cells after 28 days with intermittent CS treatment (Fig. 4b,c). The effect on MUC5AC^+^ single positive and MUC5AC^+^/SCGB1A1^+^ double positive cells was not significant. Still half of the donors showed a clear increased expression of MUC5AC (however, two donors showed a decreased number of MUC5AC^+^/SCGB1A1^−^ cells). Next, it was assessed whether CS exposure induces histological differences of the ALI cultures by performing immunohistochemical stainings of sections. Unexpectedly, CS exposed cultures as well as air controls exhibited a differentiated pseudostratified epithelium without significant differences in the epithelial thickness. However, the analysis confirmed the previous findings, that the number of SCGB1A1^+^ cells decreased in the smoke exposed ALI cultures compared to air controls, whereas the number of MUC5AC^+^ cells increased, respectively (Fig. 4d).

### Cigarette smoke exposure impairs the development of ciliated cells

**Figure 5:**
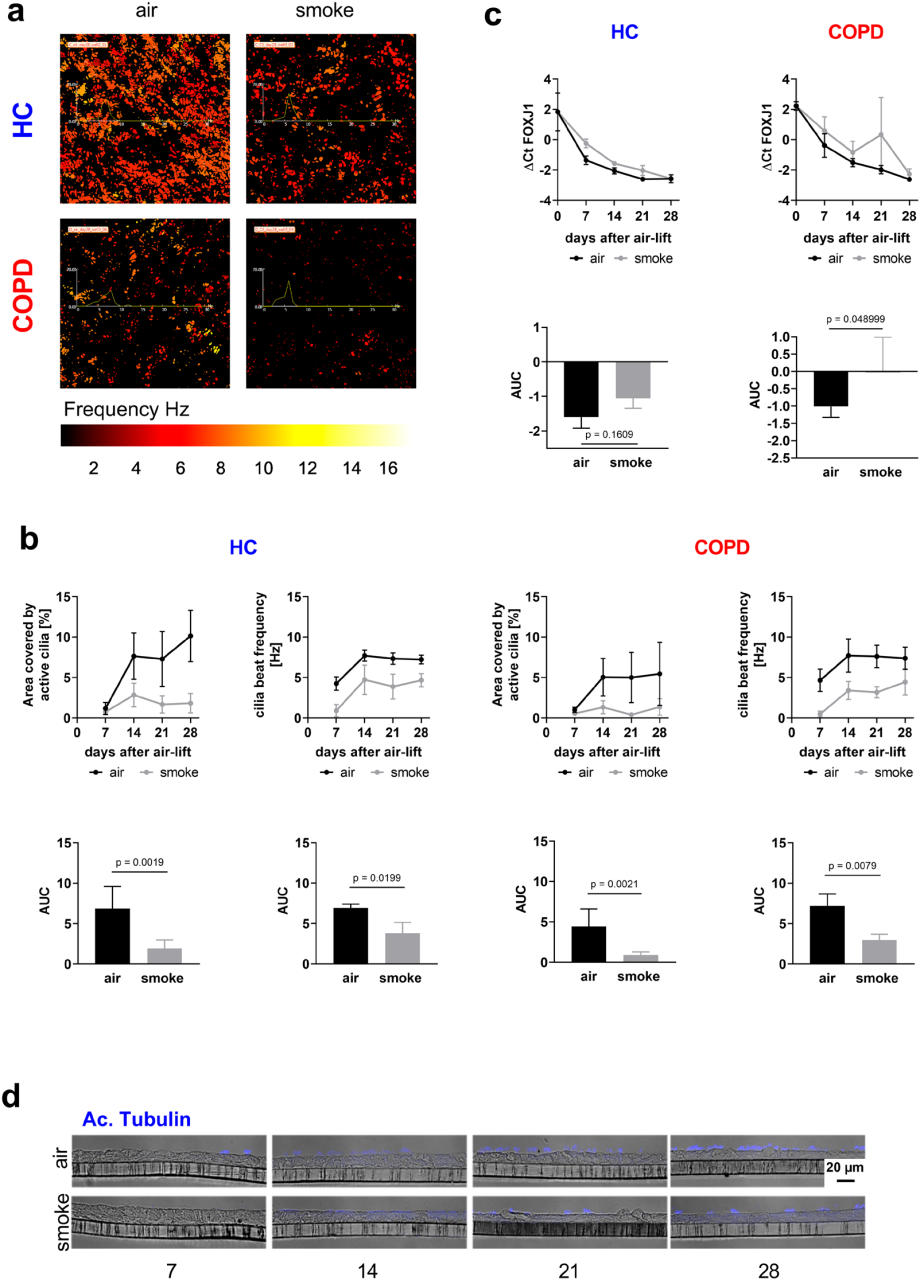
Cigarette smoke exposure impairs the development and physiology of ciliated cells. (**a**) Representative images of functional cilia beat measurement on day 28 upon air-lift of one donor with COPD and one healthy control (HC), after the exposure with CS during the differentiation process (smoke) and air treatment for control. The area covered with beating ciliated cells was visualized by false color staining and the cilia beat frequency was translated to the color code shown at the temperature lookup table at the bottom. (**b**) Quantification of the area covered by active cilia and the cilia beat frequencies at days 0, 7, 14, 21 and 28 upon air-lift. Adjusted area under the curve (AUC) was calculated to compare the development and changes over time. Data are presented as mean ± SEM. n = 3 donors with COPD and 3 healthy controls (HC); p values are based on log AUC (Area covered by active cilia) or AUC (Cilia beat frequency) values with a repeated measurement model. (**c**) Upper panel: Quantitative RT-PCR analysis of the ciliated cell marker *FOXJ1* from the same cultures. Lower panel: Adjusted area under the curve (AUC) was calculated to compare the changes of mRNA expression over time. Data are presented as mean ± SEM, p values are based on the analysis of the AUC values with a repeated measurement model. (**d**) Immunohistochemical staining of ALI sections of day 7, 14, 21 and 28 for acetylated tubulin. Representative images of one healthy donor.

The development of ciliated cells was monitored by measuring the area covered by beating cilia and their corresponding beat frequency at days 7, 14, 21 and 28. Moreover, the expression of the transcription factor *FOXJ1* and the presence of acetylated tubulin was measured, since proper cilia function, changes in transcript levels and changes in protein levels may not necessarily correspond to each other. COPD-derived cultures exhibited a delayed and reduced differentiation of ciliated cells compared to healthy cultures, indicated by a reduced area covered by actively moving cilia, and reduced acetylated tubulin expression (data not shown). These parameters were further reduced by intermittent CS treatment (Fig. 5a–c). Interestingly, cigarette smoke exposure impaired the development of ciliated cells in healthy controls in almost the same manner. To quantify the reduction in ciliary beating, the AUC was calculated and standardized with respect to the time span. Both, the area covered by actively beating cilia and the cilia beat frequency were reduced by intermittent CS treatment (Fig. 5b). *FOXJ1* mRNA increased during the normal differentiation process, indicated by a decrease in the ΔCt values (Fig. 5c). This induction was delayed in CS exposed cultures. To quantify the CS-induced effect on *FOXJ1* mRNA expression the AUC was calculated and standardized with respect to the time span (Fig. 5c). Furthermore, *FOXJ1* and *DNAI1* expression was assessed by RNA sequencing on day 28. The analysis revealed a non-significant deregulation of the ciliated cell markers on RNA level upon 28 days of intermittent CS exposure (Fig. 3c). To investigate if CS exposure not only affects the cilia function and mRNA expression but also the cell composition acetylated tubulin was stained as a cilia marker in the histological slices. Air control cultures exhibited a well-differentiated ciliated epithelium with many acetylated tubulin-positive cilia. CS-exposed ALI cultures revealed less cilia (Fig. 5d).

### CS exposure lead to the deregulation of a similar subset of genes in healthy and COPD derived ALI cultures

To elucidate the transcriptional response of the small airway epithelium to CS a NGS analysis was performed. To address the question whether COPD derived cultures are differently affected by smoking compared to healthy cultures the transcriptional response upon CS exposure was compared by performing a differential expression analysis of the NGS data. CS modulated the expression of 2442 genes in healthy cultures and 3249 genes in COPD cultures. Notably, 1945 genes were regulated in healthy as well as in COPD derived cultures. That all but one of the 1945 overlapping transcripts were regulated the same way indicates that the main stimulus of altered gene expression in the study was CS and not the disease status of the donors, i.e. COPD vs. healthy.

**Figure 6:**
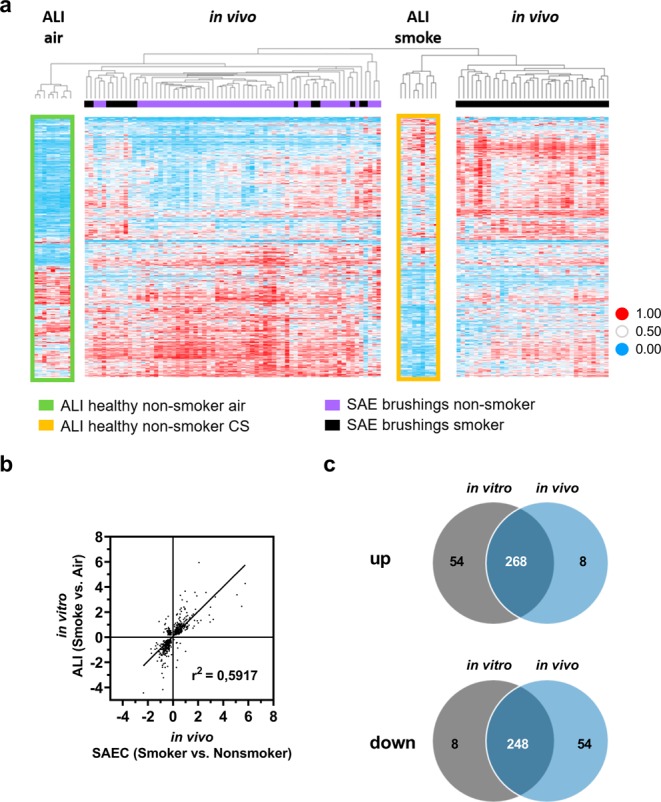
Comparison of transcriptional data from ALI experiments with *in vivo* small airway epithelium supports the translational value of the *in vitro* assay. (**a**) Heatmap representing transcripts that are significantly deregulated in SAEC ALI cultures (*in vitro* model, GSE135188) as well as in SAEC from human smokers (*in vivo* data from epithelial brushes, GSE11784) (adjusted p value <0.05 according to a linear model applied to the normalized data). Data have been independently normalized to a range between 0 and 1 on transcript level. Data sets have been joined and the resulting data set has been clustered. The gaps have been introduced into the map to visualize data representing different experiments. (**b**) Scatter plot of log ratios of the above mentioned transcripts represents the CS vs. air in ALI and smoker vs. non-smoker SAEC comparisons. (**c**) Venn diagram exhibiting the overlap within the above mentioned comparisons. Intersection represents transcripts deregulated in the same direction.

To strengthen the translational link of the *in vitro* system with the situation *in vivo*, the CS-induced transcriptional changes of the healthy ALI cultures were compared with a published data set of epithelial brushes from healthy smokers and non-smokers (GSE11784)^38^ to assess the overlap of deregulated genes. Therefore, the normalized gene expression data (range from 0 to 1) from SAE and ALI cultures were combined and filtered for those transcripts that were significantly deregulated in both comparisons: a) smoked ALI cultures versus air controls and b) SAE from healthy smokers versus SAE from non-smokers. Subsequently an unsupervised, hierarchical clustering was performed using correlation as distance measure on the combined and filtered data-set (Fig. 6a). This approach allowed the separation of different clusters according to their similarities. Two distinct clusters representing the *in vitro* samples from CS and air-treated ALI cultures were separated. Of note, most of the SAE samples from smokers clustered with the smoked ALI cultures whereas the SAE from non-smokers clustered with the ALI air-controls. This unbiased approach (which is only focusing on transcripts that were significantly deregulated but not filtering for common directionality of deregulation) indicates that CS alters overlapping molecular pathways in SAEC ALI cultures and in SAE from smokers.

**Figure 7:**
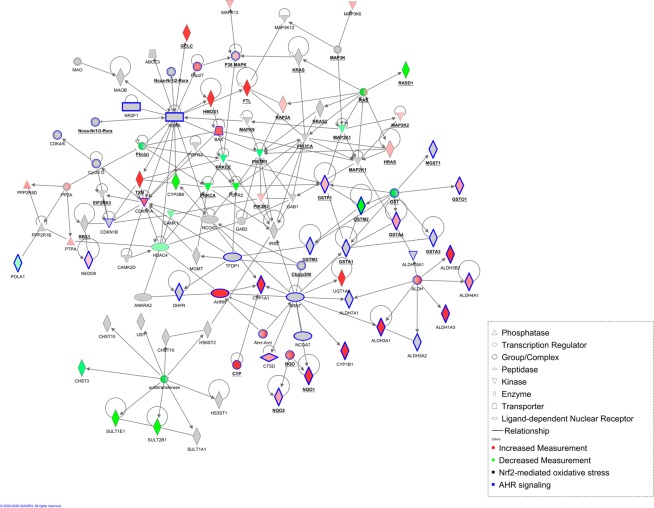
Cigarette smoke induces xenobiotic metabolism in the ALI culture as well as in smoking individuals. Network representation focusing on related transcripts (according to IPA) that are annotated as being involved in xenobiotic metabolism and are either deregulated in SAEC ALI cultures (*in vitro* model, GSE135188) or in SAEC from human smokers (*in vivo* data from epithelial brushes, GSE11784). Coloring indicates the deregulation in SAEC ALI cultures (red color = up-regulation; green = down-regulation; grey = deregulation in SAEC from human smokers only). Bold and underlined transcripts are associated with Nrf2-mediated oxidative stress; transcripts associated with AHR signaling are highlighted in blue. The network was generated through the use of IPA (QIAGEN Inc., https://www.qiagenbio-informatics.com/products/ingenuity-pathway-analysis). (Deregulation in SAEC from human smokers shown in Supplementary fig. S3).

**Figure 8:**
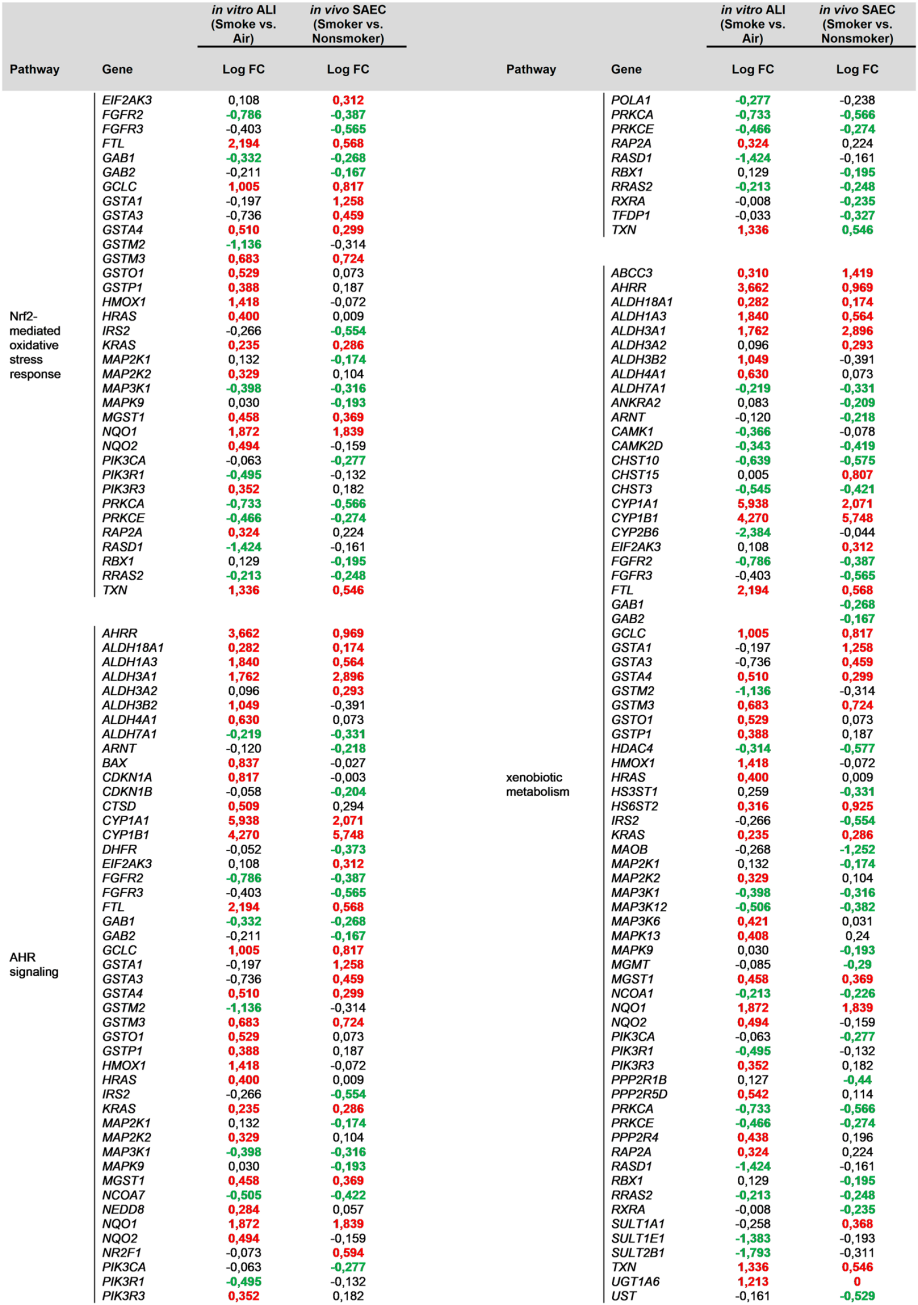
Common Cigarette Smoke Response Genes among *in vitro* and *in vivo* SAEC. Expression of Nuclear Erythroid 2-Related Factor 2 (Nrf2)-mediated oxidative stress response, Aryl Hydrocarbon Receptor (AHR) signaling and xenobiotic metabolism genes in healthy CS-exposed *in vitro* ALI cultures and *in vivo* SAEC from human smokers. Transcripts as well deregulated in SAEC ALI cultures (GSE135188) as in SAEC from human smokers (GSE11784) are listed. Red color indicates up-regulation; green indicates down-regulation; bold font indicates statistically significant deregulation (q value <0.05) according to a linear model applied to the normalized data. Association to pathways were performed according to Ingenuity Pathways Analysis.

To quantify to what extent the smoked ALI culture resembles the SAE of smokers a correlation analysis was performed (Fig. 6b). More than 86% of the genes that were significantly deregulated in the CS-exposed ALI cultures and the SAE of healthy smokers showed a consistent direction of deregulation (Fig. 6c). Furthermore, a pathway analysis was performed to elucidate the common mechanisms affected by CS-treated ALI *in vitro* and SAE from smokers. In order to allow for identifying pathways that were altered in both data sets, the significantly deregulated transcripts that exhibit the same direction of change were combined. With this set of transcripts, a core analysis was performed utilizing Ingenuity Pathways Analysis (IPA). A large number of top scoring canonical pathways were categorized to “xenobiotic metabolism” (supplementary Table S3). Therefore, all transcripts that were reported to be associated to xenobiotic metabolism were extracted and used to generate a combined network representing the *in vivo* and *in vitro* data (Fig. 7). Among the top deregulated canonical pathways associated to the network and consistent with the *in vivo* SAE data were Nuclear factor erythroid 2–related factor 2 (NRF2)-mediated oxidative stress, xenobiotic metabolism and aryl hydrocarbon (AHR) signaling (Figs. 7 and 8).

## Discussion

COPD is a disease that strongly affects the small airways^17,39^. To investigate the underlying pathophysiological and molecular mechanisms *in vitro*, a new protocol for SAEC ALI cultures and novel methods for their analysis were established. In contrast to multi-layered bronchial epithelial cell cultures, the SAEC ALI culture exhibit a two-layered pseudostratified epithelium closely resembling the human small airway *in vivo*^40^. This model allows culturing SAEC from healthy donors and COPD patients with the latter displaying some conserved pathological characteristics, such as reduced numbers of ciliated cells, increased numbers of secretory cells and a distinct gene signature. The analysis of the gene signature of the ALI cultures derived from healthy or COPD donors with *in vivo* samples from human SAE revealed a high classification power and therefore a high predictability of the health status.

A pathologic hallmark of COPD is a reduced airway epithelial barrier function leading to an increased susceptibility to infections^18–20,24–26^. In this model, CS exposure during SAEC differentiation impaired epithelial barrier formation characterized by a decreased TEER. This is consistent with previous studies investigating the effect of cigarette smoke extract on HBEC ALI cultures^27^. The observed functional decline of epithelial barrier function may be linked to the deregulation of several genes that are associated with the maintenance and control of cell-cell adhesion, permeability and tight junctions. An important molecular role in these aspects play the Claudin family members, which are tight junction membrane proteins that are conceptually divided into barrier-forming (e.g. Claudin-11, -18) and pore-forming (e.g. Claudin-7, -10) proteins^41–43^. Notably, Claudin-11 and -18, which are known to seal paracellular permeability^41^, were down-regulated in the CS exposed cultures. Claudin-18 deficiency was also described to be associated with airway epithelial barrier dysfunction and asthma^44^. Furthermore, Claudin-10, which has been unequivocally defined as pore-forming claudin^41,45^ was up-regulated. Claudin-10 and Claudin-7 were also shown to be up-regulated in smokers and COPD smokers, respectively^46^. Apart from claudins, the junctional adhesion molecule C (*JAM3*), a component of desmosomes, which mediates cell-cell adhesion^47^ was described to be down-regulated in COPD patients. *JAM3* was also decreased in the ALI cultures after 28 days of CS exposure. Taken together, different genes associated with the regulation of epithelial barrier function were deregulated in the CS-treated ALI cultures that may be associated with the pathogenesis in COPD.

Basal cell hyperplasia and squamous metaplasia play an essential role in the emergence of airway obstruction in COPD^17^ and increase the risk to develop squamous cell carcinoma^48–50^. Previous studies have found increased squamous metaplasia in the bronchial epithelium of smokers with COPD compared to healthy smokers^21^. In this study, an increase in several genes associated with squamous metaplasia, namely *KRT5*, *KRT6A*, *KRT13*, *KRT14* and *SFN* was observed indicating that CS plays a role in squamous differentiation. These *in vitro* findings are in accordance with histological (known for a long time^51^) and expression analyses (more recent ones^52^) of COPD patients indicating that basal cells are the first cell population affected in smokers and leading to an altered differentiation pattern and architecture of the small airway epithelium. Taken together, the model can be used to investigate the molecular mechanisms underlying the early effects of smoking on basal cell proliferation.

Smoking induces changes in secretory cell populations leading to hyperplasia of mucous-producing goblet cells and a reduction of club cells, which are associated with epithelial defense mechanisms^7,53^. These findings resemble the goblet cell hyperplasia in COPD patients, which was shown to be associated with current smoking^6,54^. Recent findings indicate a shift in the differentiation pattern of the small airways of smokers and COPD patients towards a more proximal airway epithelium^35,55^; an effect recently described as smoking-dependent distal-to-proximal repatterning^56^. Goblet cells for example were found in the small airways of smokers^6^, where they are normally absent. This aspect of the disease was found in the smoked SAEC ALI cultures. CS exposure led to a reduction of club cell markers (SCGB1A1, CYP2F1) and a downward trend of MUC5AC^−^/SCGB1A1^+^ cells in the ALI cultures. This may reflect the pathophysiological decline in club cells observed in COPD. The reduced club cell numbers in the airways of smokers and COPD patients, respectively^7,15^, are associated with reduced uteroglobin (SCGB1A1) serum levels, which were correlated to a decline in FEV1 (Forced Expiratory Volume in 1 Second) and COPD disease progression^14,52^. In contrast to our findings, the exposure to CSE did not show a reduction in club cell numbers^27^. Therefore, we think that the apical exposure of the SAEC ALI culture with whole CS leads to a better representation of the pathophysiological processes induced in human smokers. The model may help to dissect the molecular mechanisms, leading to the impaired differentiation of basal cells in COPD patients^14,53,57–59^.

Coordinated and efficient ciliary beating is essential to clear the airways from inhaled particles and pathogens and is therefore an important defense mechanism^60^. COPD patients often suffer from severe exacerbations caused by bacterial or viral infections, due to insufficient clearance^26,61^. We show that CS exposure during SAEC differentiation impairs the development and function of ciliated cells. These findings are consistent with studies, investigating the effect of cigarette smoke extract on human bronchial epithelial cell differentiation^27^. To evaluate to which extent the CS ALI *in vitro* model reflects smoking induced effects on the lung epithelium of small airways in living humans, a transcriptomic comparison analysis of SAEC ALI cultures and human SAE brushings was conducted. Of note, SAEC ALI cultures stimulated with CS exhibit a gene expression pattern overlapping to epithelial samples from healthy smokers. When combining both datasets and performing a hierarchical clustering on the overlapping de-regulated transcripts, it would be expected to see similar patterns but not to find the *in vivo* data and the *in vitro* data forming separate clusters. Remarkably, CS stimulated SAEC ALI cultures clustered with samples from smokers, whereas air treated SAEC ALI cultures clustered with healthy control samples in an unexpected fashion. By this means, we did not include mechanisms that were only affected *in vitro* and not *in vivo* or vice versa, but we focused on processes that were modulated in both situations. Our results highlight the presence of shared processes that show a corresponding de-regulation, i.e. a similar up or downregulation *in vitro* and *in vivo*. Thus, we think that the CS treated ALI culture from SAEC is a valuable *in vitro* method to investigate smoke induced effects on epithelial differentiation. However, our data does not allow distinguishing CS-induced effects in ALI cultures from healthy donors compared to COPD patients, since the CS is a very dominant driver of differential changes. The dissection of potential differences in smoked healthy versus smoked COPD ALI cultures would require a much higher donor number. Here we see a clear limitation of our complex experimental *in vitro* setup; nevertheless, ALI cultures from COPD patients may be extremely helpful to test whether potential therapeutic concepts work also in a diseased setting.

Among the top deregulated canonical pathways were Nuclear factor erythroid 2–related factor 2 (NRF2)-mediated oxidative stress, xenobiotic metabolism and aryl hydrocarbon receptor (AHR) signaling^62–65^. It is known that CS induces reactive oxygen species, which activates the NRF2 pathway^63,66^. Moreover, CS comprises a number of toxic chemicals including polycyclic aromatic hydrocarbons (PAH)^67^. A prominent member of PAH is benzo[a]pyrene, which activates the AHR pathway, inducing the expression of several metabolic enzymes, including *CYP1A1*, *CYP1A2* and *CYP1B1*^62,68^. The model reflects these CS induced pathways in close resemblance to the *in vivo* situation and offers novel opportunities to study new therapeutic targets addressing oxidative stress responses and xenobiotic metabolism.

Inconsistencies with the *in vivo* data were strongly associated with lipid metabolism (e.g. synthesis of cholesterol).

Our data demonstrate that CS specifically alters the small airway epithelium by affecting basal cell differentiation. Thereby, important pathways are consistently modulated compared to the small airways of human smokers. Taken together, critical pathophysiologic and molecular effects could be mimicked *in vitro* that were described in small airways of smokers, which might lead to novel essential therapeutic approaches to restore the impaired epithelial repair mechanisms in a smoke-induced chronic disease like COPD.

## Methods

### SAEC ALI culture

Human Small Airway Epithelial Cells (SAEC, CC-2547, Lonza, Basel, Switzerland) of three healthy donors and three COPD patients (see Supplementary Table S1) were cultured as described in Supplementary Information. As stated by Lonza, the cells were isolated from donated human tissue after obtaining permission for their use in research applications by informed consent or legal authorization. Established ethical practices of the donation and transplantation organizations in the US (American Association of Tissue Banks, Association of Organ Procurement Organizations, Eye Bank Association of America) are followed at Lonza. All methods were carried out in accordance with relevant guidelines and regulations. Human SAEC were exclusively used for *in vitro* experiments. SAEC were grown in PneumaCult™-Ex Plus Medium (Stemcell Technologies, Vancouver, Canada) on rat tail collagen type 1 (Corning Life Sciences B.V., Amsterdam, the Netherlands) coated transwells (#3460, Corning Life Sciences B.V., Amsterdam, the Netherlands) until confluent. Cells were further cultivated in air-liquid interface using Pneumacult-ALI-S Medium (Stemcell Technologies, Vancouver, Canada) for four weeks until fully differentiated.

### Cigarette smoke exposure

SAECs were exposed to whole cigarette smoke of K3R4F reference cigarettes (University of Kentucky, Lexington, KY, USA) using an automated cigarette smoking machine (In-Expose smoking robot, Scireq Montreal, QC, Canada) and the P.R.I.T. (Professional *In-Vitro* Technologies) ExpoCube in the experimental setup developed by Fraunhofer Institute for Toxicology and Experimental Medicine. Four cigarettes were smoked in parallel in compliance to ISO 3308, drawing every 15 seconds a puff from the sequentially smoked cigarettes (9 puffs per cigarette). The smoke was diluted with ambient air (dilution rate: 0.5 l/min). The cells were exposed to CS three times a week during the differentiation phase (28 days) beginning on day 0 (= day of air-lift). Cells were cultured on 12-well plates with the upper four wells exposed to smoke and the lower four wells exposed to air^69^. All wells were uniformly exposed to CS due to computational fluid dynamics-optimized geometry of the flow paths. This was previously tested and described in detail by Ritter *et al*., Fraunhofer Institute for Toxicology and Experimental Medicine^70^. Readouts were performed 24 hours post last exposure.

Cytotoxicity of smoke-exposed cells was assessed on day 28, based on the measurement of lactate dehydrogenase (LDH) activity released from the cytosol of damaged cells into the supernatant (compare Supplementary Methods). There was no significant difference in LDH release between smoke-exposed and air-exposed cells (Supplementary Fig. S4).

### Transepithelial Electrical Resistance (TEER)

Epithelial barrier integrity was monitored by measuring Transepithelial Electrical Resistance (TEER) using an EVOM2 epithelial volt-ohmmeter (World precision Instruments, Sarasota, FL, USA) according to the manufacturer’s instructions. To perform the TEER measurement, phosphate buffered saline (PBS, 300 µl) was added to the apical compartment of the insert. The long stem of the electrode was inserted through the gap of the transwell to be in contact with the basolateral medium and the short stem of the electrode was placed above the apical surface to be in contact with the apical PBS. The measured resistance was multiplied by the surface area of the epithelium (1.12 cm^2^) to obtain TEER (Ω*cm^2^).

### Histology and Immunohistochemistry

Cells, growing on transwell membranes (pore size of 0.4 µm), were fixed in 4% paraformaldehyde over night and embedded in paraffin. After sectioning (section slice thickness approximately 3–4 µm), slides were deparaffinized and hematoxylin and eosin (H&E)  stainings were performed according to standard procedures. For immunohistochemical (IHC) investigation, paraffin-embedded tissue slides were subjected to heat induced antigen retrieval in citrate buffer (pH 6.0, H.I.E.R, BioLegend San Diego, CA, USA) for 30 minutes. Antibodies used for immunofluorescence or chromogenic antigen detection are listed in Supplementary Information, Table S2. After staining, tissues slides were mounted either with ProLong™ Diamond Antifade Mountant (Thermo Fisher Scientific, Waltham, MA, USA) or with Entellan® new (Merck KGaA, Darmstadt, Germany). For immunofluorescence samples, stained slides were stored at 4 °C in the dark and images were taken using the Zeiss Laser Scanning Microscope 710 or an AxioImager M2 (both Carl Zeiss, Oberkochen, Germany) in case of chromogenic IHC. Stainings for KRT5, MUC5AC, SCGB1A1 and acetylated Tubulin were used to assess protein expression qualitatively and representatively. Claudin-10 protein expression was semi-quantitatively analyzed using digital image analysis (compare section “Image Analysis”).

### Image Analysis

ALI cultures with Claudin-10 IHC staining (ab52234, Rabbit polyclonal to Claudin-10, Abcam, Cambridge, MA, USA) were scanned with an Axio Scan.Z1 scanner (Carl Zeiss Microscopy GmbH, Jena, Germany) using a 20x objective (0.22 µm/px) in bright field illumination. Semi-quantitative image analysis was performed with the Halo 3.0 software using the CytoNuclear 1.6 module (Indicalabs, Corales, NM, USA). In preparation for the automated analysis, regions with damaged ALIs or insufficiently stained cells were manually excluded. For automated analysis, a random forest tissue classifier was trained to recognize the cellular layer of the ALIs to limit the following analysis to this region and exclude the supporting material and background. Subsequently, the CytoNuclear module was applied within this region. As first step, the channels of nuclear (hematoxylin) and IHC stain (3’Diaminobenzidine (DAB) signal of Claudin-10) were unmixed using color deconvolution. Cell segmentation used the nuclear stain and empirical parameters on size and shape of the cells. Positive cells were identified using a manually optimized threshold value on the DAB channel, within the segmented region of a cell. The generated readout per ALI culture was the number of positive cells n per analyzed area of the cell layer A, i.e. n/A.

### Cilia beat measurement of SAEC ALI cultures

Ciliary function was analyzed using video-microscopy as described in Supplementary Information. Briefly, cilia beat frequency was calculated from image stacks of 2D + time. For each pixel in the field of view, the signal in time domain is searched for a meaningful frequency. The results were mapped back to image space forming an image, where each pixel represents the beat frequency of the cilia covering that area.

### RT-qPCR

RT-qPCRs were conducted using the High-Capacity cDNA Reverse Transcription Kit and TaqMan Assay-on-Demand kits from Applied Biosystems (Darmstadt, Germany) for *KRT5* (Hs00361185_m1), *MUC5AC* (Hs01365616_m1), *SCGB1A1* (Hs00171092_m1), *FOXJ1* (HS00230964_m1), *KRT14* (Hs00265033_m1) and *POLR2A* (Hs00172187_m1). The relative expression of the RNAs was determined by the comparative Ct method. Expression values were normalized to the control RNA (*POLR2A*).

### Next generation sequencing and detection of differentially expressed genes

RNA extraction, Illumina library preparation and sequencing was performed as previously described^71^. Comparative analysis was done using the limma R-package^72^ by applying a the following linear model:

anno.sub < - data.frame(group = pre.design$group,donorID = pre.design$donorID)

dmat < - model.matrix(~0+group+donorID,data = anno.sub)

Benjamini-Hochberg correction was used to adjust for multiple testing. Expression data have been deposited to Gene Expression Omnibus (https://www.ncbi.nlm.nih.gov/geo/; accession no. GSE135188). The previously published data set GSE11784^38^ was used for comparative analysis.

### Hierarchical clustering

Hierarchical clustering was performed with TIBCO Spotfire Analyst 7.11.1 LTS HF-013. As clustering method UPGM with correlation as distance measure was used for both columns and rows, respectively. Expression values were normalized to a per transcript range between 0 and 1 prior to clustering.

### Classification

The Random Forest classifier offered by WEKA Workbench 3.8.3 (Weka, University of Waikato, New Zealand) with parameters -P 100 -I 100 -num-slots 1 -K 10 -M 1.0 -V 0.001 -S 1 was used for classifying samples and generating ROC curves and confusion matrices. Classification was performed on expression values normalized to a per transcript range between 0 and 1. The WEKA CorrelationAttributeEval attribute evaluator (Weka, University of Waikato, New Zealand) with standard parameters and WEKA Ranker as search method with standard parameters was used for ranking features according to their classification power.

### Network generation

Ingenuity Pathway Analysis (IPA; Qiagen) version 48207413 was used to perform all network related analyses. Molecules associated with “Xenobiotic Metabolism Signaling” were compiled, utilizing the “Pathways and Tox Lists” related search tool, and gathered in one pathway view. Subsequently, molecules were overlaid with ALI (GSE135188) and SAE (GSE11784) expression data representing significantly deregulated transcripts in one or the other experiment. Molecules, which did not show deregulation in either experiment, were removed. The “Connect” tool was used to introduce relationships as reported within the IPA knowledge base among the remaining transcripts. Orphans and smaller graphs (less than four nodes) were removed in order to focus on the largest interconnected graph. To focus on specific transcripts and increase readability, a number of hubs were removed from the graph. Finally, either ALI or SAE expression values were laid over the resulting network and transcripts representing selected biological processes according to the IPA knowledge base were highlighted.

### Flow cytometry

Single cell suspensions of SAEC ALI cultures (see Supplementary Information) were fixed in 4% paraformaldehyde for 20 minutes at room temperature. Cells were washed twice using Perm/Wash Buffer (BD Biosciences, San Jose, CA, USA) and blocked to prevent non-specific binding using Human BD Fc Block (BD Biosciences, San Jose, CA, USA). Cells were stained in Perm/Wash Buffer for 1 hour at room temperature. Anti-human antibodies used for staining are listed in the supplement. Flow cytometry was performed using a BD LSRFortessa X-20 cytometer equipped with DIVA-software (BD Biosciences, San Jose, CA, USA).

### Statistics

Statistical testing used for RNA sequencing analysis is described in section “Next generation sequencing and detection of differentially expressed genes”. All the other analyses were performed as followed. Instead of modelling the longitudinal data, the analyses were performed with adjusted areas under the curve (AUC) or the logarithmized adjusted AUC. The adjusted AUC was obtained by dividing the AUC by the length of the considered time interval. Each of the endpoints was analyzed separately. The data was analyzed with a repeated measurement model, as both treatments (CS/Air) were applied to cells of each donor. Compound symmetry was assumed as underlying covariance structure in order to account for the repeated measurement within a donor. The fixed factors status of the cells (HC/COPD), treatment (CS/Air) and the interaction of status of the cells and treatment were included in the model. The degrees of freedom were calculated by dividing the residual degrees of freedom into between-subject and within-subject portions. If a fixed effect changed within any subject, the within-subject degrees of freedom were assigned to the considered fixed effect (cell status, treatment, interaction effect). Otherwise, the between-subject degrees of freedom were used. The applied repeated measurement model assumes that the errors follow a normal distribution.

Using linear combinations of the parameter estimates obtained from the repeated measurement model, it was tested whether treatment with CS compared to Air had an impact on the considered endpoint. This analysis was performed separately for each status of cells (HC/COPD). Depending on the expected change of the endpoint under the treatment with smoke, the following hypotheses were tested:

- ${ {\mathcal H} }_{0,{\rm{HC}},{\rm{Lower}}}:{\mu }_{{\rm{HC}},{\rm{CS}}}-{\mu }_{{\rm{HC}},{\rm{Air}}}\ge 0$ vs. ${ {\mathcal H} }_{1,{\rm{Lower}}}:{\mu }_{{\rm{HC}},{\rm{CS}}}-{\mu }_{{\rm{HC}},{\rm{Air}}} < 0$ and ${ {\mathcal H} }_{0,{\rm{COPD}},{\rm{Lower}}}:{\mu }_{{\rm{COPD}},{\rm{CS}}}-$ ${\mu }_{{\rm{COPD}},{\rm{Air}}}\ge 0$ vs. ${{\mathcal{H}}}_{1,{\rm{L}}{\rm{o}}{\rm{w}}{\rm{e}}{\rm{r}}}:{\mu }_{{\rm{C}}{\rm{O}}{\rm{P}}{\rm{D}},{\rm{C}}{\rm{S}}}-{\mu }_{{\rm{C}}{\rm{O}}{\rm{P}}{\rm{D}},{\rm{A}}{\rm{i}}{\rm{r}}} < 0$, where *μ*_*i,j*_ denotes the mean value of the investigated endpoint under treatment *j* for the status of cells *i*.
  For the following endpoints the above stated hypotheses were tested as a decrease was assumed when cells were treated with smoke: Logarithmized AUC of Area covered by active cilia, AUC of Cilia beat frequency, logarithmized AUC of TEER, AUC of KRT5 mRNA (Ct), AUC of KRT14 mRNA (Ct), AUC of MUC5AC mRNA (Ct).
- ${ {\mathcal H} }_{0,{\rm{HC}},{\rm{Upper}}}:{\mu }_{{\rm{HC}},{\rm{CS}}}-{\mu }_{{\rm{HC}},{\rm{Air}}}\le 0$ vs. ${ {\mathcal H} }_{1,{\rm{HC}},{\rm{Upper}}}:{\mu }_{{\rm{HC}},{\rm{CS}}}-{\mu }_{{\rm{HC}},{\rm{Air}}} > 0$ and ${ {\mathcal H} }_{0,{\rm{COPD}},{\rm{Upper}}}:{\mu }_{{\rm{COPD}},{\rm{CS}}}-$ ${\mu }_{{\rm{COPD}},{\rm{Air}}}\le 0$ vs. ${ {\mathcal H} }_{1,{\rm{COPD}},{\rm{Upper}}}:{\mu }_{{\rm{COPD}},{\rm{CS}}}-{\mu }_{{\rm{COPD}},{\rm{Air}}} > 0.$

For the following endpoints the above stated hypotheses were tested as an increase was assumed when cells were treated with smoke: Claudin-10 protein expression, AUC of SCGB1A1 mRNA (Ct), AUC of FOXJ1 mRNA (Ct).

All tests are t-test like tests based on the linear combinations of the parameter estimates from the repeated measurement model and were performed post-hoc. The p-values were not adjusted for multiplicity induced by the number of tested endpoints and hypotheses and the selection of the finally applied statistical model.

The statistical evaluation was prepared using the software package SAS Version 9.4 (SAS Institute Inc., Cary, North Carolina, USA).

## Supplementary information

Supplementary Information.

## Data Availability

All relevant data are within the paper and its Supplemental Information file. Sequencing data that support the findings of this study have been deposited in Gene Expression Omnibus (GEO; https://www.ncbi.nlm.nih.gov/geo/) with the accession code GSE135188.
