# Supplementary material for: Intermittent exposure to whole cigarette smoke alters the differentiation of primary small airway epithelial cells in the air-liquid interface culture
Source: Sci Rep. 2020 Apr 10;10:6257. doi: 10.1038/s41598-020-63345-5 (PMC7148343; doi:10.1038/s41598-020-63345-5)
Supplement: Supplementary file 1 — Supplementary Information. [file 41598_2020_63345_MOESM1_ESM.pdf]

Supplementary Information

Intermittent exposure to whole cigarette smoke alters the differentiation of primary small airway epithelial cells in the air-liquid interface culture

Authors:

Julia A. Gindele[1,2], Tobias Kiechle[1], Kerstin Benediktus[1], Gerald Birk[3], Michael Brendel[4], Fabian Heinemann[3], Christian T. Wohnhaas[5,6], Michelle LeBlanc[7], Haijun Zhang[7], Yael Strulovici-Barel[7], Ronald G. Crystal[7], Matthew J. Thomas[1], Birgit Stierstorfer[3], Karsten Quast[5], Jürgen Schymeinsky[1,2,*]

[1]Immunology & Respiratory Diseases Research, Boehringer Ingelheim Pharma GmbH & Co. KG, Biberach an der Riß, Germany

[2]Department of General Physiology, University of Ulm, Ulm, Germany

[3]Drug Discovery Sciences, Boehringer Ingelheim Pharma GmbH & Co. KG, Biberach an der Riß, Germany

[4]Non-Clinical Statistics Biberach, Boehringer Ingelheim Pharma GmbH & Co. KG, Biberach an der Riß, Germany

[5]Global Computational Biology and Digital Sciences, Boehringer Ingelheim Pharma GmbH & Co. KG, Biberach an der Riß, Germany

[6]Department of Biology, University of Konstanz, Konstanz, Germany

[7]Department of Genetic Medicine, Weill Cornell Medical College, New York, NY 10065

* juergen.schymeinsky@boehringer-ingelheim.com

Table S1: SAEC Donor Information

	Age (years)	Sex	Race	Smoking
HC-1	67	Female	Caucasian	No
HC-2	42	Female	Hispanic	No
HC-3	38	Male	Caucasian	No
COPD-1	57	Female	Caucasian	Yes
COPD-2	51	Female	Caucasian	Yes
COPD-3	62	Female	Black	Yes

SAEC ALI culture

Human Small Airway Epithelial Cells (SAEC, CC-2547, Lonza, Basel, Switzerland) were thawed and approximately one million cells were seeded into a T175 cell culture flask with 50 ml pre-warmed PneumaCult™-Ex Plus Medium (Stemcell Technologies, Vancouver, Canada). On the following day, the medium was exchanged, to remove residuals from the cryoprotectant cocktail. On the fourth day after seeding, the cells were sufficiently confluent (max. 80 %) to be harvested for seeding onto transwell inserts. The Transwells (#3460, Corning Life Sciences B.V., Amsterdam, the Netherlands) were coated with 300 µl rat tail collagen type 1 solution (Corning Life Sciences B.V., Amsterdam, the Netherlands, 30µg/ml in phosphate buffered saline) for 45 minutes at 37 °C. Collagen solution was aspirated and transwell inserts were washed with phosphate buffered saline. The basolateral compartments of the transwells were filled with 1.5 ml PneumaCult™-Ex Plus Medium. Dissociation of SAEC was performed using the Animal Component-Free Cell Dissociation Kit (Stemcell Technologies, Vancouver, Canada) according to the manufacturer's instructions. Subsequently, 100.000 cells per insert were seeded in 300 µl of PneumaCult™-Ex Plus Medium on the transwell membrane. On the following day, apical and basolateral media was exchanged using PneumaCult™-Ex Plus Medium. On day three after seeding onto transwell inserts, cells were fully confluent. The apical and basolateral medium was removed and 1.5 ml Pneumacult-ALI-S Medium (Stemcell Technologies, Vancouver, Canada) was added basolaterally. The apical compartment was washed with phosphate buffered saline to remove residual growth factors of the expansion medium. Afterwards cell were maintained in air-liquid interface (day 0) to allow differentiation into pseudostratified epithelial cultures. Medium exchanges were performed three times a week. The cells were fully differentiated 28 days post air-lift.

Preparation of a single cell suspension from SAEC ALI cultures

Fully differentiated SAEC ALI cultures growing on 12 well transwells inserts were dissociated enzymatically and mechanically. Cells were incubated with 300 µl Accumax solution (Sigma-Aldrich, St. Louis, MO, USA) for 15 minutes at room temperature. Apical liquid containing already dissociated cells was transferred to a tube and 300 µl fresh Accumax solution were added into the transwell insert for another 15 minutes. By clapping the plate gently, cells dissociated from the membrane. Transwell membrane was washed with Accumax solution several times until cell layer was completely dissociated. Cells were transferred into a gentleMACS C Tube and were gently dissociated by the gentleMACS Dissociator (Miltenyi Biotec, Bergisch Gladbach, Germany) using programme m_lung_01. The suspension was centrifuged and resuspended in 0.04% Bovine Serum Albumin in Hank's Balanced Salt Solution. This step was repeated several times to obtain a single cell suspension. Large aggregates were removed by passing the suspension through 30 µm cell strainers. Cell concentration, viability and number of aggregates were determined using a NucleoCounter NC-200 (ChemoMetec, Allerod, Denmark).

Cilia Beat Measurement

A variety of methods are used to obtain quantitative data from beating cilia, these range from manual or automated [1,2] tracking of cilia in video sequences to very sophisticated solutions with optical coherence tomography [3].

Repeated observations of vital cell cultures on air-liquid interfaces limit the options for data acquisition. We chose to use video-microscopy. Unfortunately, the ciliary beat frequency cannot be directly extracted from the image space. Manual tracking is a time consuming task, fully automated processes would ease this workload, but there appear to be challenges in the ability to incorporate intensity, resolution and focal changes. What we are interested in is the beat frequency, and this is in time domain [4]. However, in a video of beating cilia, the time domain is not directly accessible. To circumvent this, we use image stacks of 2D+time, 512 images with a size of 512 *512 pixels. If the microscopic images describe the transversal planes, then sagittal and coronal planes nicely present the intensity modulated signal caused by beating cilia.

The disadvantage is that an image in a sagittal or coronal plane still has time and spatial information (Fig. S1a). A power spectrum generated from such an image would be difficult to interpret, however, an image from a single line of size 1 pixel by 512 pixels, simplifies the analysis. A line profile of this image with intensity changes caused by a beating cilia would also allow simple counting of swings per time as shown in Figure S1b (39 beats in 512 frames at 100 fps result in beat frequency of 7.62 Hz) or could be sent to any other Fourier analysis tool.

We implemented a fully automated version using imaging processing tools, including the Discrete Fourier Transformation, for the characterization of a ciliary beating pattern and frequency in an air-liquid interface cell culture. The corresponding time track is taken for all the pixels in the x-y (spatial) domain. The signal is multiplied by a Gaussian window function

before the fast Fourier transformation is calculated. The resulting power spectrum is searched for the most relevant frequency and the result is mapped back onto x-y-coordinates (Fig. S1c).

The results for all pixels are represented using a false-color lookup table (LUT), in this example a temperature LUT, i.e. the faster the hotter (Figure S1d). A histogram of frequencies [Hz] found for the field of view is displayed as an overlay. The mean frequency, area covered by moving cilia, and histogram of frequencies present in the experiment are stored on file. The analysis of an image stack takes approximately one minute. Time could be further reduced by optimizing code for reading and rotating volume, using parallelization and minimizing stack size if required.

Typical beat frequencies are in the range of 5 Hz to 15 Hz. Analyzing beating cilia with a minimum of 50 frames per second would theoretically be sufficient, but we prefer to use a camera running at 100 frames per second (acA 1300-200µm, black and white, USB-3.0, Basler AG, 22926 Ahrensburg, Germany). A stack size of the power of two is not mandatory but often used in the field of medical imaging and is often preferred when using Fourier transformations with image processing tools. Applications for image capture and analysis are developed using a commercially available machine vision software toolbox (Halcon 13.0.2, MVTec Software GmbH, Munich). Visualization of image stack was done with Analyze (AnalyzeDirect, Inc. Overland Park, KS, USA).

Figure S1

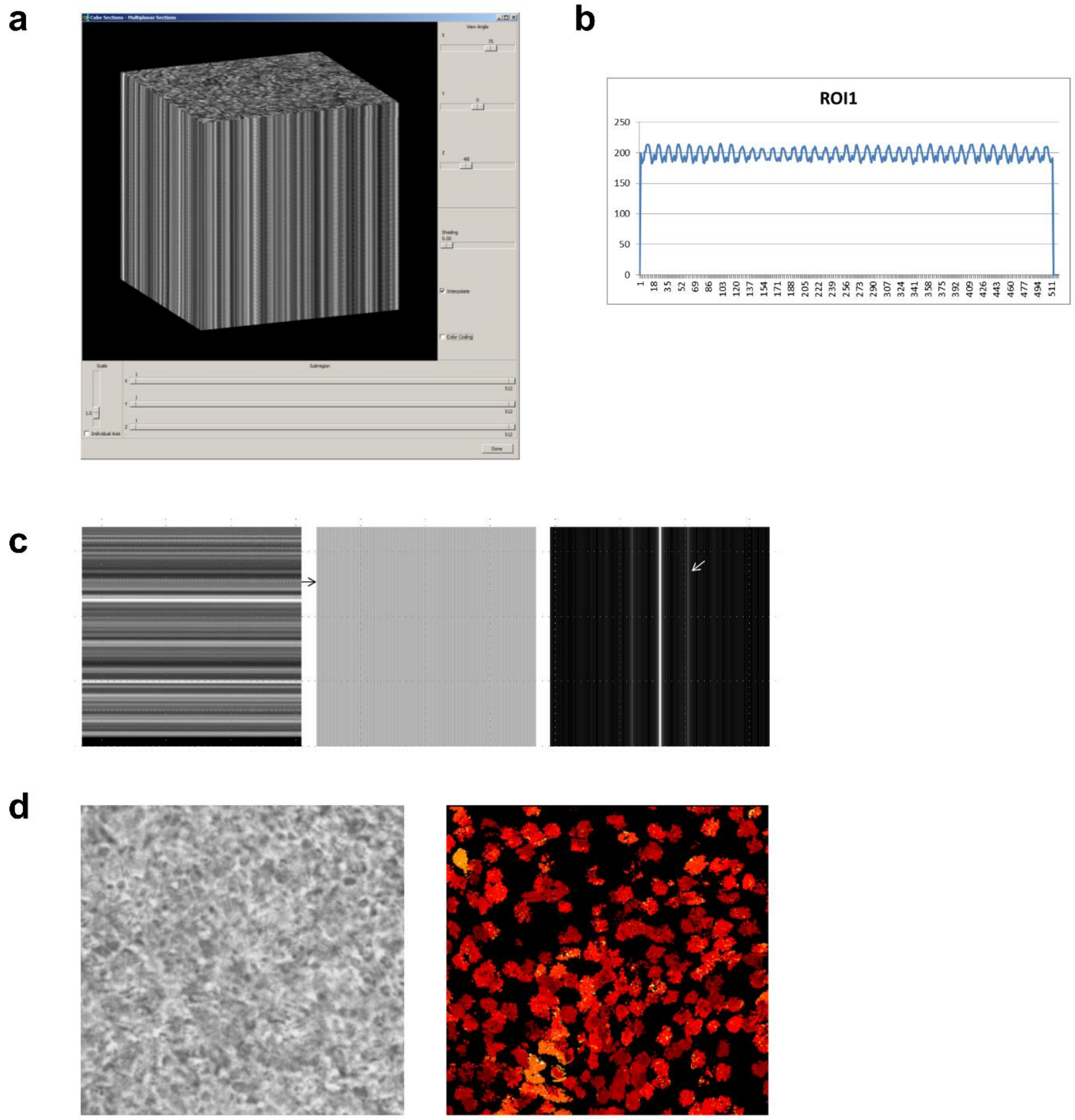

Quantitative cilia beat measurement. (a) 2D+t stack of a SAEC culture at air liquid interface. (b) Intensity changes over time taken from a single line of voxels of a 2D+t stack. (c) Image of 1D+time (left), single line image (middle, stretched to 512 by 512 pixels) and the corresponding fast Fourier transform (right) where the significant frequency can be seen. (d) One image out of the image stack (left) and the frequency image (right).

Table S2: Antibodies for Immunohistofluorescence and Flow Cytometry

Application	Antigen	Antibody
IHC	Cytokeratin 5 (KRT5)	rabbit monoclonal Anti-Cytokeratin 5 antibody, clone EP1601Y, ab193895 (Abcam, Cambrige, UK)
	Mucin 5 AC (MUC5AC)	mouse monoclonal Anti-MUC5AC antibody, clone 45M1, NBP2-32732AF488 (Novus Biologicals, Centennial, CO, USA)
	Uteroglobin (SCGB1A1)	rat monoclonal Anti-SCGB1A1 antibody, Clone #394324, MAB4218 (R&D; Minneapolis, MN, USA)
	Acteylated Tubulin (Ac. Tub.)	Mouse monoclonal Anti-Acetylated Tubulin antibody, clone 6-11B-1 (Sigma-Aldrich, St. Louis, MO, USA)
	Secondary antibody	secondary Goat anti-Rat IgG (H+L) Cross-Adsorbed Antibody, Alexa Fluor 568, A-11077 (Life Technologies, Oregon, OR, USA)
	Claudin 10 (CLDN10)	Rabbit polyclonal Anti-Claudin 10 antibody, ab52234 (Abcam, Cambrige, UK)
FACS	Cytokeratin 5 (KRT5)	Rabbit monoclonal Anti-Cytokeratin 5 antibody, clone EP1601Y, ab193895 (Abcam, Cambrige, UK)
	Mucin 5 AC (MUC5AC)	mouse monoclonal Anti-MUC5AC antibody, clone 45M1, NBP2-32732AF405 (Novus Biologicals, Centennial, CO, USA)
	Uteroglobin (SCGB1A1)	rat monoclonal Anti-SCGB1A1 antibody, Clone #394324, MAB4218 (R&D; Minneapolis, MN, USA)
	Secondary antibody	Mouse monoclonal Anti-Rat IgG1 secondary antibody, PE, Clone R1-12D10, 12-4812-82 (eBioscience, San Diego, CA, USA)

Figure S2

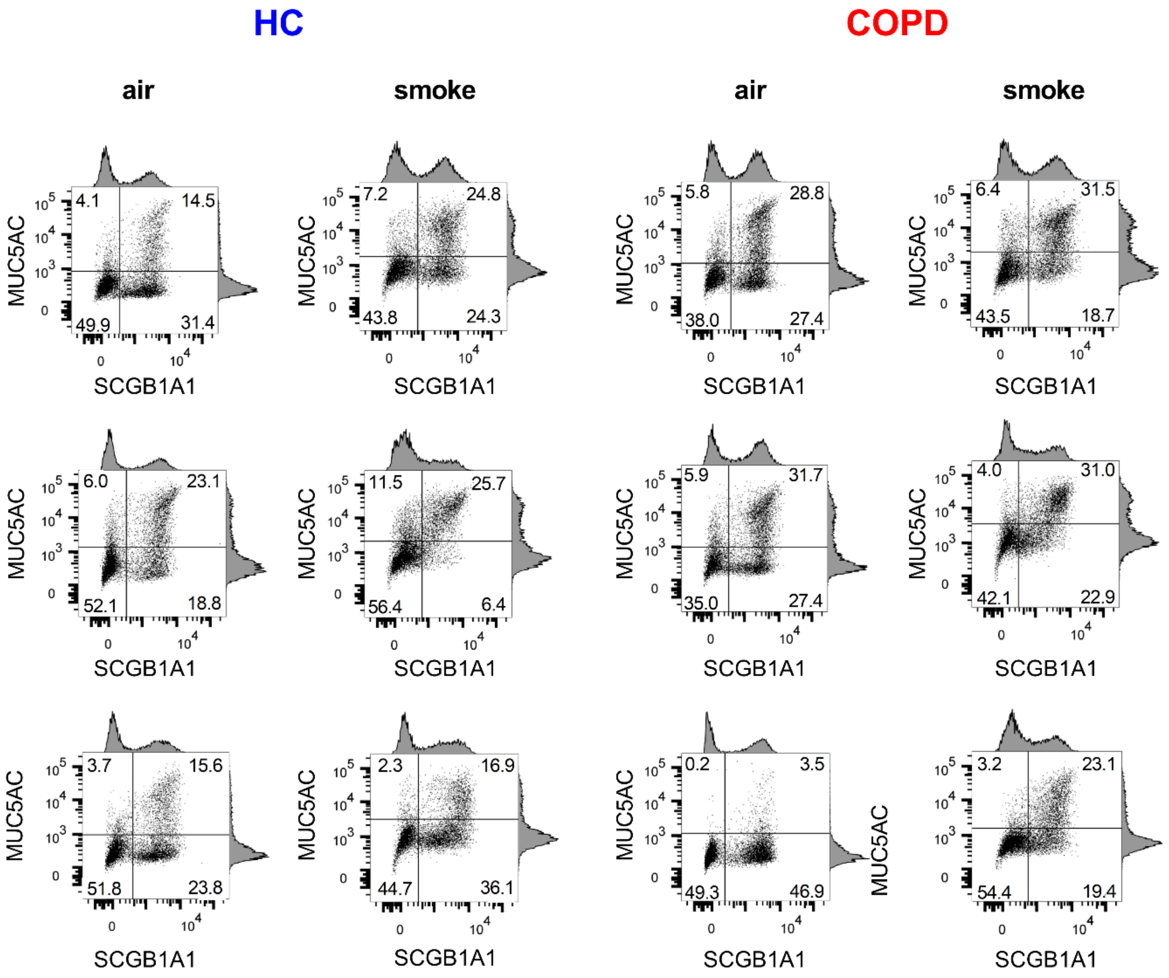

FACS analysis of the secretory cell marker MUC5AC and SCGB1A1. Scatterplot of FACS analysis. The data were generated at day 28 of ALI culture upon air-lift. The cultures were treated with intermittent exposure of CS or air for control. n = 3 donors with COPD and 3 healthy controls (HC).

Figure S3

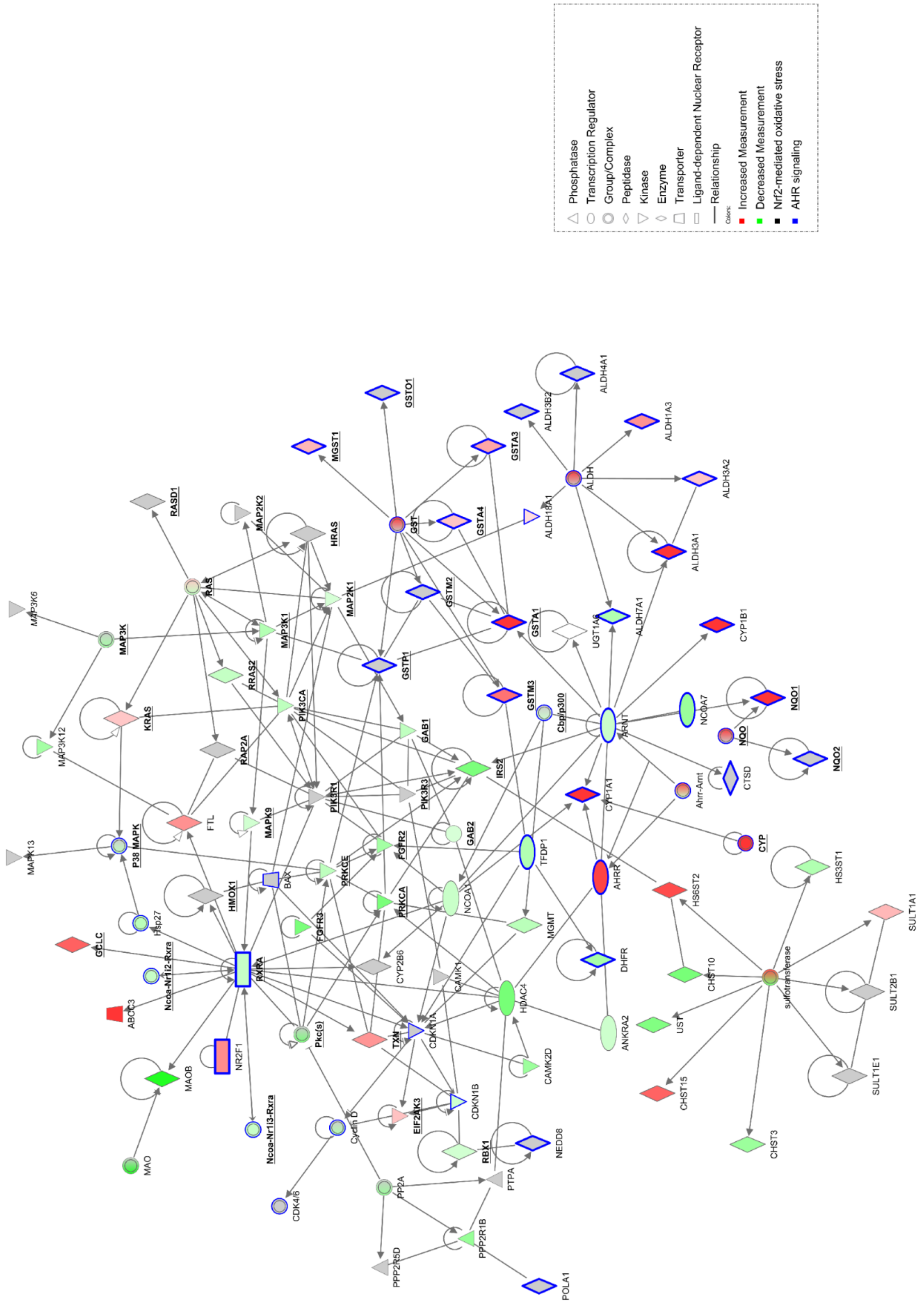
Phosphatase
Transcription Regulator
Group/Complex
Peptidase
Kinase
Enzyme
Transporter
Ligand-dependent Nuclear Receptor
Relationship
Colors:
Increased Measurement
Decreased Measurement
Nrf2-mediated oxidative stress
AHR signaling
ALDH4A1
GSTO1
ALDH3B2
ALDH1A3
MGST1
GSTA3
ALDH
ALDH3A2
RASD1
MAP2K2
HRAS
GST
GSTA4
ALDH18A1
ALDH3A1
RAS
MAP2K1
GSTM2
MAP3K6
MAP3K
MAP3K1
GSTA1
UGT1A6
ALDH7A1
CYP1B1
GSTP1
RRAS2
PIK3CA
KRAS
GSTM3
Cbp/p300
ARNT
NCOA7
NQO1
NQO
NQO2
MAP3K12
GAB1
RAP2A
IRS2
CTSD
PIK3R1
PIK3R3
CYP1A1
Ahr-Arnt
MAPK13
P38 MAPK
FTL
MAPK9
GAB2
CYP
FGFR2
TFDP1
HS3ST1
BAX
PRKCE
AHRR
HMOX1
NCOA1
HS6ST2
SULT1A1
Hsp27
PRKCA
FGFR3
MGMT
GCLC
Ncoa-Nr1i2-Rxra
RXRA
CYP2B6
DHFR
CHST10
sulfotransferase
CAMK1
SULT2B1
ABCC3
HDAC4
TXN
CDKN1A
ANKRA2
UST
Pkc(s)
NR2F1
MAOB
CAMK2D
CHST15
SULT1E1
EIF2AK3
CDKN1B
Ncoa-Nr1i3-Rxra
Cyclin D
MAO
RBX1
NEDD8
CHST3
CDK4/6
PTPA
PP2A
PPP2R1B
PPP2R5D
POLA1

Cigarette smoke induces xenobiotic metabolism in the ALI culture as well as in smoking inividuals. Network representation focusing on related transcripts (according to IPA) that are annotated as being involved in xenobiotic metabolism and are either de-regulated in SAEC ALI cultures (*in vitro* model) or in SAEC from human smokers (*in vivo* data from epithelial brushes). Coloring indicates the deregulation in SAEC from human smokers (red color = up-regulation; green = down-regulation; grey = de-regulation in SAEC ALI cultures only). Bold and underlined transcripts are associated with Nrf2-mediated oxidative stress; transcripts associated with AHR signaling are highlighted in blue. The network was generated through the use of IPA (QIAGEN Inc., https://www.qiagenbioinformatics.com/products/ingenuity-pathway-analysis).

Table 3: Deregulated Canonical Pathways according to Ingenuity Pathways Analysis

Ingenuity Canonical Pathways	-log(p-value)	Ratio
NRF2-mediated Oxidative Stress Response	7,95	0,14
Estrogen Biosynthesis	4,77	0,22
Xenobiotic Metabolism Signaling	4,05	0,09
Bupropion Degradation	3,58	0,24
Pentose Phosphate Pathway	3,46	0,40
RAR Activation	3,44	0,09
γ-glutamyl Cycle	3,28	0,36
Protein Kinase A Signaling	3,26	0,07
Nicotine Degradation II	3,16	0,14
Acetone Degradation I (to Methylglyoxal)	3,12	0,20
LPS/IL-1 Mediated Inhibition of RXR Function	3,08	0,08
Aryl Hydrocarbon Receptor Signaling	3,00	0,10
Acute Phase Response Signaling	2,92	0,09
Retinoate Biosynthesis I	2,90	0,18
Hepatic Fibrosis Signaling Pathway	2,79	0,07
STAT3 Pathway	2,76	0,10
Vitamin-C Transport	2,60	0,25
Methylglyoxal Degradation III	2,40	0,22
Glutathione Biosynthesis	2,39	0,67
Nicotine Degradation III	2,32	0,13
Melatonin Signaling	2,26	0,11
Iron homeostasis signaling pathway	2,26	0,09
cAMP-mediated signaling	2,24	0,07
GPCR-Mediated Integration of Enteroendocrine Signaling Exemplified by an L Cell	2,23	0,11
Glutathione-mediated Detoxification	2,20	0,16
Antioxidant Action of Vitamin C	2,10	0,09
Pentose Phosphate Pathway (Oxidative Branch)	2,10	0,50
Pregnenolone Biosynthesis	1,94	0,23
Glutathione Redox Reactions I	1,94	0,17
Complement System	1,93	0,14
Tyrosine Degradation I	1,89	0,40
Osteoarthritis Pathway	1,86	0,07
Colanic Acid Building Blocks Biosynthesis	1,85	0,21
Role of Macrophages. Fibroblasts and Endothelial Cells in Rheumatoid Arthritis	1,84	0,06
G-Protein Coupled Receptor Signaling	1,83	0,07
Ovarian Cancer Signaling	1,80	0,08
Aldosterone Signaling in Epithelial Cells	1,79	0,08
Histidine Degradation VI	1,77	0,20
Cardiac Hypertrophy Signaling (Enhanced)	1,76	0,06
ERK5 Signaling	1,74	0,10
Pentose Phosphate Pathway (Non-oxidative Branch)	1,72	0,33
Sperm Motility	1,67	0,07
Ubiquinol-10 Biosynthesis (Eukaryotic)	1,62	0,18
Histamine Degradation	1,62	0,18
Melatonin Degradation I	1,61	0,10
TNFR2 Signaling	1,61	0,13
Thioredoxin Pathway	1,59	0,29
Autophagy	1,58	0,10
Wnt/Ca+ pathway	1,55	0,10
Agranulocyte Adhesion and Diapedesis	1,53	0,07
Colorectal Cancer Metastasis Signaling	1,53	0,06
Ethanol Degradation II	1,51	0,13
p53 Signaling	1,51	0,08
Oxidative Ethanol Degradation III	1,49	0,16
Chondroitin Sulfate Biosynthesis (Late Stages)	1,48	0,10
Superoxide Radicals Degradation	1,47	0,25
PXR/RXR Activation	1,46	0,09
Superpathway of Melatonin Degradation	1,46	0,09
Leukocyte Extravasation Signaling	1,45	0,07
The Visual Cycle	1,43	0,15
MIF-mediated Glucocorticoid Regulation	1,43	0,12
Role of JAK2 in Hormone-like Cytokine Signaling	1,43	0,12
Xanthine and Xanthosine Salvage	1,43	1,00
Adenine and Adenosine Salvage VI	1,43	1,00
UDP-N-acetyl-D-galactosamine Biosynthesis I	1,43	1,00
Granulocyte Adhesion and Diapedesis	1,42	0,07
NF-κB Signaling	1,42	0,07
TR/RXR Activation	1,42	0,08
TNFR1 Signaling	1,41	0,10
Cardiac β-adrenergic Signaling	1,40	0,07
TWEAK Signaling	1,39	0,11
Noradrenaline and Adrenaline Degradation	1,39	0,11
Maturity Onset Diabetes of Young (MODY) Signaling	1,37	0,14
UDP-N-acetyl-D-galactosamine Biosynthesis II	1,37	0,22
Corticotropin Releasing Hormone Signaling	1,33	0,07
Hepatic Fibrosis / Hepatic Stellate Cell Activation	1,32	0,06
Pancreatic Adenocarcinoma Signaling	1,28	0,07
Ethanol Degradation IV	1,27	0,13
p70S6K Signaling	1,27	0,07
Death Receptor Signaling	1,26	0,08
Glioma Signaling	1,26	0,07
Caveolar-mediated Endocytosis Signaling	1,25	0,08
Relaxin Signaling	1,25	0,07
Type I Diabetes Mellitus Signaling	1,24	0,07
Inhibition of Matrix Metalloproteases	1,24	0,10
Chondroitin Sulfate Biosynthesis	1,23	0,09
Leptin Signaling in Obesity	1,23	0,08
GNRH Signaling	1,21	0,06
Wnt/β-catenin Signaling	1,21	0,06
PI3K/AKT Signaling	1,19	0,06
Tryptophan Degradation X (Mammalian. via Tryptamine)	1,18	0,12
Toll-like Receptor Signaling	1,18	0,08
Dermatan Sulfate Biosynthesis	1,15	0,08
Gap Junction Signaling	1,15	0,06
Role of Osteoblasts. Osteoclasts and Chondrocytes in Rheumatoid Arthritis	1,14	0,06
Gluconeogenesis I	1,14	0,12
Guanosine Nucleotides Degradation III	1,14	0,17
Guanine and Guanosine Salvage I	1,13	0,50
L-DOPA Degradation	1,13	0,50

Alanine Degradation III	1,13	0,50
Alanine Biosynthesis II	1,13	0,50
Sulfate Activation for Sulfonation	1,13	0,50
Adenine and Adenosine Salvage I	1,13	0,50
GDP-L-fucose Biosynthesis I (from GDP-D-mannose)	1,13	0,50
Glutamate Dependent Acid Resistance	1,13	0,50
Oncostatin M Signaling	1,12	0,09
tRNA Splicing	1,12	0,09
Apelin Cardiomyocyte Signaling Pathway	1,10	0,07
Role of IL-17A in Psoriasis	1,08	0,15
Urate Biosynthesis/Inosine 5'-phosphate Degradation	1,08	0,15
NAD Phosphorylation and Dephosphorylation	1,08	0,15
Bile Acid Biosynthesis. Neutral Pathway	1,08	0,15
Neuropathic Pain Signaling In Dorsal Horn Neurons	1,07	0,07
Hepatic Cholestasis	1,06	0,06
Apelin Adipocyte Signaling Pathway	1,06	0,07
IL-17A Signaling in Airway Cells	1,04	0,08
Glioblastoma Multiforme Signaling	1,03	0,06
Dermatan Sulfate Biosynthesis (Late Stages)	1,03	0,09
PFKFB4 Signaling Pathway	1,03	0,09
HER-2 Signaling in Breast Cancer	1,02	0,07
Dopamine Degradation	1,00	0,10
PDGF Signaling	0,98	0,07
P2Y Purigenic Receptor Signaling Pathway	0,98	0,06
Choline Biosynthesis III	0,97	0,13
Adenosine Nucleotides Degradation II	0,97	0,13
Uracil Degradation II (Reductive)	0,97	0,33
NADH Repair	0,97	0,33
Tetrahydrobiopterin Biosynthesis I	0,97	0,33
Thymine Degradation	0,97	0,33
Tetrahydrobiopterin Biosynthesis II	0,97	0,33
S-adenosyl-L-methionine Biosynthesis	0,97	0,33
Tyrosine Biosynthesis IV	0,97	0,33
Gαs Signaling	0,97	0,07
Role of NFAT in Cardiac Hypertrophy	0,96	0,06
Regulation of the Epithelial-Mesenchymal Transition Pathway	0,96	0,06
RANK Signaling in Osteoclasts	0,95	0,07
Adrenomedullin signaling pathway	0,91	0,06
Circadian Rhythm Signaling	0,90	0,09
3-phosphoinositide Degradation	0,90	0,06
GPCR-Mediated Nutrient Sensing in Enteroendocrine Cells	0,89	0,06
D-myo-inositol-5-phosphate Metabolism	0,89	0,06
D-myo-inositol (1.4.5)-trisphosphate Degradation	0,88	0,12
CD27 Signaling in Lymphocytes	0,86	0,08
Non-Small Cell Lung Cancer Signaling	0,86	0,07
Arsenate Detoxification I (Glutaredoxin)	0,85	0,25
α-tocopherol Degradation	0,85	0,25
Glutathione Redox Reactions II	0,85	0,25
Proline Biosynthesis I	0,85	0,25
Phenylalanine Degradation I (Aerobic)	0,85	0,25
Acetate Conversion to Acetyl-CoA	0,85	0,25
Purine Nucleotides Degradation II (Aerobic)	0,84	0,11
PI3K Signaling in B Lymphocytes	0,83	0,06
EGF Signaling	0,82	0,07
Interferon Signaling	0,82	0,08
Insulin Receptor Signaling	0,82	0,06
ATM Signaling	0,80	0,06
Bladder Cancer Signaling	0,80	0,06
Hereditary Breast Cancer Signaling	0,80	0,06
D-myo-inositol (1.4.5.6)-Tetrakisphosphate Biosynthesis	0,80	0,06
D-myo-inositol (3.4.5.6)-tetrakisphosphate Biosynthesis	0,80	0,06
Macropinocytosis Signaling	0,80	0,07
Unfolded protein response	0,80	0,07
B Cell Receptor Signaling	0,80	0,05
Thrombin Signaling	0,80	0,05
Role of NANOG in Mammalian Embryonic Stem Cell Pluripotency	0,80	0,06
Notch Signaling	0,79	0,08
Type II Diabetes Mellitus Signaling	0,78	0,06
Apoptosis Signaling	0,78	0,06
IL-15 Production	0,77	0,06
Fatty Acid α-oxidation	0,76	0,10
Protein Citrullination	0,76	0,20
Tetrahydrofolate Salvage from 5.10-methenyltetrahydrofolate	0,76	0,20
Serine Biosynthesis	0,76	0,20
Tetrapyrrole Biosynthesis II	0,76	0,20
Lysine Degradation II	0,76	0,20
Myo-inositol Biosynthesis	0,76	0,20
Lysine Degradation V	0,76	0,20
Galactose Degradation I (Leloir Pathway)	0,76	0,20
Glutamate Degradation III (via 4-aminobutyrate)	0,76	0,20
April Mediated Signaling	0,75	0,08
RhoA Signaling	0,75	0,06
Tight Junction Signaling	0,74	0,05
Huntington's Disease Signaling	0,74	0,05
Putrescine Degradation III	0,73	0,10
Prolactin Signaling	0,73	0,06
Clathrin-mediated Endocytosis Signaling	0,72	0,05
Phagosome Formation	0,72	0,06
NF-κB Activation by Viruses	0,71	0,06
FXR/RXR Activation	0,71	0,06
Induction of Apoptosis by HIV1	0,71	0,07
PTEN Signaling	0,71	0,06
IGF-1 Signaling	0,71	0,06
Glucocorticoid Receptor Signaling	0,71	0,05
B Cell Activating Factor Signaling	0,70	0,07
Mechanisms of Viral Exit from Host Cells	0,70	0,07
Triacylglycerol Biosynthesis	0,70	0,07
Proline Biosynthesis II (from Arginine)	0,69	0,17
Chondroitin and Dermatan Biosynthesis	0,69	0,17
Arginine Degradation VI (Arginase 2 Pathway)	0,69	0,17
UDP-N-acetyl-D-glucosamine Biosynthesis II	0,69	0,17
Adenine and Adenosine Salvage III	0,69	0,17
Glycogen Biosynthesis II (from UDP-D-Glucose)	0,69	0,17

GDP-mannose Biosynthesis	0,69	0,17
Superpathway of Inositol Phosphate Compounds	0,68	0,05
Phagosome Maturation	0,68	0,05
MIF Regulation of Innate Immunity	0,68	0,07
Intrinsic Prothrombin Activation Pathway	0,68	0,07
Retinol Biosynthesis	0,68	0,07
FGF Signaling	0,68	0,06
Synaptic Long Term Potentiation	0,68	0,05
Thrombopoietin Signaling	0,68	0,06
Phospholipases	0,68	0,06
Superpathway of D-myo-inositol (1.4.5)-trisphosphate Metabolism	0,67	0,09
IL-4 Signaling	0,67	0,06
Opioid Signaling Pathway	0,66	0,05
IL-8 Signaling	0,66	0,05
Cellular Effects of Sildenafil (Viagra)	0,65	0,05
Pyridoxal 5'-phosphate Salvage Pathway	0,64	0,06
IL-23 Signaling Pathway	0,64	0,07
Superpathway of Serine and Glycine Biosynthesis I	0,63	0,14
Phosphatidylcholine Biosynthesis I	0,63	0,14
Purine Ribonucleosides Degradation to Ribose-1-phosphate	0,63	0,14
HGF Signaling	0,62	0,05
iNOS Signaling	0,62	0,07
IL-17A Signaling in Gastric Cells	0,62	0,08
Role of JAK family kinases in IL-6-type Cytokine Signaling	0,62	0,08
Role of Oct4 in Mammalian Embryonic Stem Cell Pluripotency	0,61	0,07
eNOS Signaling	0,61	0,05
Dendritic Cell Maturation	0,60	0,05
NAD Salvage Pathway II	0,59	0,08
Glycolysis I	0,59	0,08
IL-1 Signaling	0,59	0,05
Th17 Activation Pathway	0,59	0,05
SPINK1 General Cancer Pathway	0,59	0,06
Apelin Endothelial Signaling Pathway	0,58	0,05
Histidine Degradation III	0,58	0,13
Salvage Pathways of Pyrimidine Deoxyribonucleotides	0,58	0,13
Dopamine-DARPP32 Feedback in cAMP Signaling	0,57	0,05
Neuroprotective Role of THOP1 in Alzheimer's Disease	0,57	0,05
Endothelin-1 Signaling	0,56	0,05
Production of Nitric Oxide and Reactive Oxygen Species in Macrophages	0,56	0,05
3-phosphoinositide Biosynthesis	0,56	0,05
Growth Hormone Signaling	0,56	0,06
Small Cell Lung Cancer Signaling	0,56	0,06
Sphingosine-1-phosphate Signaling	0,56	0,05
Role of Tissue Factor in Cancer	0,56	0,05
Heparan Sulfate Biosynthesis (Late Stages)	0,56	0,06
Cell Cycle: G2/M DNA Damage Checkpoint Regulation	0,55	0,06
Renin-Angiotensin Signaling	0,55	0,05
Synaptogenesis Signaling Pathway	0,55	0,04
PPARα/RXRα Activation	0,55	0,05
α-Adrenergic Signaling	0,55	0,05
Basal Cell Carcinoma Signaling	0,55	0,06
GABA Receptor Signaling	0,55	0,05
Amyloid Processing	0,54	0,06
GP6 Signaling Pathway	0,54	0,05
Sucrose Degradation V (Mammalian)	0,54	0,11
Heme Biosynthesis II	0,54	0,11
Folate Transformations I	0,54	0,11
Neuregulin Signaling	0,54	0,05
Sonic Hedgehog Signaling	0,53	0,07
UVC-Induced MAPK Signaling	0,52	0,06
Salvage Pathways of Pyrimidine Ribonucleotides	0,52	0,05
Molecular Mechanisms of Cancer	0,52	0,04
Estrogen-Dependent Breast Cancer Signaling	0,52	0,05
Glioma Invasiveness Signaling	0,52	0,05
Hypoxia Signaling in the Cardiovascular System	0,52	0,05
UVA-Induced MAPK Signaling	0,51	0,05
G Beta Gamma Signaling	0,51	0,05
UVB-Induced MAPK Signaling	0,51	0,06
Nitric Oxide Signaling in the Cardiovascular System	0,50	0,05
Embryonic Stem Cell Differentiation into Cardiac Lineages	0,50	0,10
Prostanoid Biosynthesis	0,50	0,10
Erythropoietin Signaling	0,50	0,05
G Protein Signaling Mediated by Tubby	0,49	0,06
Dopamine Receptor Signaling	0,49	0,05
Role of IL-17A in Arthritis	0,48	0,06
VDR/RXR Activation	0,47	0,05
Heparan Sulfate Biosynthesis	0,47	0,05
Neuroinflammation Signaling Pathway	0,47	0,04
Mouse Embryonic Stem Cell Pluripotency	0,46	0,05
Sumoylation Pathway	0,46	0,05
Epithelial Adherens Junction Signaling	0,46	0,05
Role of BRCA1 in DNA Damage Response	0,45	0,05
White Adipose Tissue Browning Pathway	0,45	0,05
Gustation Pathway	0,45	0,05
T Cell Receptor Signaling	0,45	0,05
CNTF Signaling	0,44	0,05
LPS-stimulated MAPK Signaling	0,43	0,05
PEDF Signaling	0,43	0,05
MSP-RON Signaling Pathway	0,43	0,05
Virus Entry via Endocytic Pathways	0,43	0,05
CREB Signaling in Neurons	0,43	0,04
Coagulation System	0,42	0,06
CDK5 Signaling	0,42	0,05
Adipogenesis pathway	0,41	0,04
Semaphorin Signaling in Neurons	0,41	0,05
Assembly of RNA Polymerase III Complex	0,41	0,08
Sertoli Cell-Sertoli Cell Junction Signaling	0,41	0,04
Oleate Biosynthesis II (Animals)	0,41	0,08
Leukotriene Biosynthesis	0,41	0,08
PCP pathway	0,41	0,05
AMPK Signaling	0,40	0,04
Cell Cycle Regulation by BTG Family Proteins	0,39	0,05
Cardiac Hypertrophy Signaling	0,38	0,04

Synaptic Long Term Depression	0,38	0,04
Tec Kinase Signaling	0,38	0,04
Regulation of IL-2 Expression in Activated and Anergic T Lymphocytes	0,37	0,04
Endocannabinoid Developing Neuron Pathway	0,36	0,04
Androgen Biosynthesis	0,36	0,07
CD40 Signaling	0,35	0,05
Myc Mediated Apoptosis Signaling	0,35	0,05
Fc Epsilon RI Signaling	0,35	0,04
Protein Ubiquitination Pathway	0,35	0,04
Endocannabinoid Cancer Inhibition Pathway	0,35	0,04
Calcium-induced T Lymphocyte Apoptosis	0,34	0,05
Eicosanoid Signaling	0,34	0,05
p38 MAPK Signaling	0,34	0,04
Axonal Guidance Signaling	0,34	0,04
Role of PKR in Interferon Induction and Antiviral Response	0,34	0,05
Granzyme B Signaling	0,34	0,06
Extrinsic Prothrombin Activation Pathway	0,34	0,06
Glutaryl-CoA Degradation	0,34	0,06
Pyrimidine Ribonucleotides Interconversion	0,34	0,05
Parkinson's Signaling	0,34	0,06
Systemic Lupus Erythematosus In B Cell Signaling Pathway	0,34	0,04
Serotonin Degradation	0,34	0,04
Cell Cycle: G1/S Checkpoint Regulation	0,34	0,04
Factors Promoting Cardiogenesis in Vertebrates	0,33	0,04
Cholecystokinin/Gastrin-mediated Signaling	0,33	0,04
Melanocyte Development and Pigmentation Signaling	0,33	0,04
Role of IL-17F in Allergic Inflammatory Airway Diseases	0,33	0,05
ErbB Signaling	0,33	0,04
LXR/RXR Activation	0,32	0,04
Dermatan Sulfate Degradation (Metazoa)	0,32	0,06
IL-10 Signaling	0,32	0,04
Role of JAK1 and JAK3 in γc Cytokine Signaling	0,32	0,04
Pyrimidine Ribonucleotides De Novo Biosynthesis	0,32	0,05
Serotonin Receptor Signaling	0,32	0,05
1D-myo-inositol Hexakisphosphate Biosynthesis II (Mammalian)	0,30	0,06
D-myo-inositol (1.3.4)-trisphosphate Biosynthesis	0,30	0,06
FAT10 Signaling Pathway	0,30	0,06
CCR3 Signaling in Eosinophils	0,30	0,04
Ephrin B Signaling	0,29	0,04
Calcium Signaling	0,29	0,04
VEGF Signaling	0,29	0,04
Atherosclerosis Signaling	0,29	0,04
RhoGDI Signaling	0,29	0,04
GADD45 Signaling	0,29	0,05
DNA damage-induced 14-3-3σ Signaling	0,29	0,05
14-3-3-mediated Signaling	0,28	0,04
T Helper Cell Differentiation	0,28	0,04
Role of Pattern Recognition Receptors in Recognition of Bacteria and Viruses	0,28	0,04
Endocannabinoid Neuronal Synapse Pathway	0,28	0,04
nNOS Signaling in Neurons	0,28	0,04
CDP-diacylglycerol Biosynthesis I	0,27	0,05
Inflammasome pathway	0,27	0,05
SAPK/JNK Signaling	0,27	0,04
GDNF Family Ligand-Receptor Interactions	0,26	0,04
Neurotrophin/TRK Signaling	0,26	0,04
PPAR Signaling	0,26	0,04
HOTAIR Regulatory Pathway	0,26	0,04
Role of Wnt/GSK-3β Signaling in the Pathogenesis of Influenza	0,25	0,04
IL-7 Signaling Pathway	0,25	0,04
Phosphatidylglycerol Biosynthesis II (Non-plastidic)	0,25	0,05
Methionine Degradation I (to Homocysteine)	0,25	0,05
IL-3 Signaling	0,24	0,04
Role of Lipids/Lipid Rafts in the Pathogenesis of Influenza	0,23	0,04
Differential Regulation of Cytokine Production in Intestinal Epithelial Cells by IL-17A and IL-17F	0,23	0,04
Tryptophan Degradation III (Eukaryotic)	0,23	0,04
Apelin Cardiac Fibroblast Signaling Pathway	0,23	0,04
Lymphotoxin β Receptor Signaling	0,23	0,04
Role of JAK1. JAK2 and TYK2 in Interferon Signaling	0,22	0,04
Cysteine Biosynthesis III (mammalia)	0,22	0,04
D-myo-inositol (1.4.5)-Trisphosphate Biosynthesis	0,21	0,04
Lipid Antigen Presentation by CD1	0,20	0,04
Antiproliferative Role of TOB in T Cell Signaling	0,20	0,04
Estrogen-mediated S-phase Entry	0,20	0,04
Natural Killer Cell Signaling	0,00	0,03
Amyotrophic Lateral Sclerosis Signaling	0,00	0,03
Actin Cytoskeleton Signaling	0,00	0,03
Mitochondrial Dysfunction	0,00	0,03
Ceramide Signaling	0,00	0,02
Regulation of Actin-based Motility by Rho	0,00	0,03
Activation of IRF by Cytosolic Pattern Recognition Receptors	0,00	0,02
Fcγ Receptor-mediated Phagocytosis in Macrophages and Monocytes	0,00	0,03
IL-12 Signaling and Production in Macrophages	0,00	0,03
Role of RIG1-like Receptors in Antiviral Innate Immunity	0,00	0,02
TREM1 Signaling	0,00	0,03
Role of NFAT in Regulation of the Immune Response	0,00	0,02
CCR5 Signaling in Macrophages	0,00	0,03
Cytotoxic T Lymphocyte-mediated Apoptosis of Target Cells	0,00	0,03
IL-17 Signaling	0,00	0,03
fMLP Signaling in Neutrophils	0,00	0,03
CXCR4 Signaling	0,00	0,04
4-1BB Signaling in T Lymphocytes	0,00	0,03
CTLA4 Signaling in Cytotoxic T Lymphocytes	0,00	0,02
IL-9 Signaling	0,00	0,03
CD28 Signaling in T Helper Cells	0,00	0,03
IL-15 Signaling	0,00	0,01
Role of Cytokines in Mediating Communication between Immune Cells	0,00	0,02
Reelin Signaling in Neurons	0,00	0,03
HIF1α Signaling	0,00	0,03
Angiopoietin Signaling	0,00	0,01
Agrin Interactions at Neuromuscular Junction	0,00	0,03
Docosahexaenoic Acid (DHA) Signaling	0,00	0,03
iCOS-iCOSL Signaling in T Helper Cells	0,00	0,02
Mitotic Roles of Polo-Like Kinase	0,00	0,02

HMGB1 Signaling	0,00	0,02
Role of CHK Proteins in Cell Cycle Checkpoint Control	0,00	0,02
FLT3 Signaling in Hematopoietic Progenitor Cells	0,00	0,01
Human Embryonic Stem Cell Pluripotency	0,00	0,04
Antiproliferative Role of Somatostatin Receptor 2	0,00	0,03
Androgen Signaling	0,00	0,03
Germ Cell-Sertoli Cell Junction Signaling	0,00	0,03
Melanoma Signaling	0,00	0,02
Prostate Cancer Signaling	0,00	0,03
Renal Cell Carcinoma Signaling	0,00	0,04
Acute Myeloid Leukemia Signaling	0,00	0,01
Thyroid Cancer Signaling	0,00	0,02
Graft-versus-Host Disease Signaling	0,00	0,02
Chronic Myeloid Leukemia Signaling	0,00	0,03
Gα12/13 Signaling	0,00	0,03
mTOR Signaling	0,00	0,03
Communication between Innate and Adaptive Immune Cells	0,00	0,02
Crosstalk between Dendritic Cells and Natural Killer Cells	0,00	0,03
Systemic Lupus Erythematosus Signaling	0,00	0,01
Cdc42 Signaling	0,00	0,01
ILK Signaling	0,00	0,04
FAK Signaling	0,00	0,02
EIF2 Signaling	0,00	0,01
Retinoic acid Mediated Apoptosis Signaling	0,00	0,02
PAK Signaling	0,00	0,02
Rac Signaling	0,00	0,04
Phospholipase C Signaling	0,00	0,04
Altered T Cell and B Cell Signaling in Rheumatoid Arthritis	0,00	0,02
Breast Cancer Regulation by Stathmin1	0,00	0,04
Nur77 Signaling in T Lymphocytes	0,00	0,03
PKCθ Signaling in T Lymphocytes	0,00	0,03
Role of MAPK Signaling in the Pathogenesis of Influenza	0,00	0,04
Role of PI3K/AKT Signaling in the Pathogenesis of Influenza	0,00	0,03
Role of Hypercytokinemia/hyperchemokinemia in the Pathogenesis of Influenza	0,00	0,02
OX40 Signaling Pathway	0,00	0,03
Inhibition of Angiogenesis by TSP1	0,00	0,03
Cyclins and Cell Cycle Regulation	0,00	0,04
Cell Cycle Control of Chromosomal Replication	0,00	0,04
Assembly of RNA Polymerase II Complex	0,00	0,02
IL-17A Signaling in Fibroblasts	0,00	0,03
Actin Nucleation by ARP-WASP Complex	0,00	0,01
NGF Signaling	0,00	0,04
Paxillin Signaling	0,00	0,01
Signaling by Rho Family GTPases	0,00	0,03
Telomerase Signaling	0,00	0,03
Transcriptional Regulatory Network in Embryonic Stem Cells	0,00	0,04
nNOS Signaling in Skeletal Muscle Cells	0,00	0,02
VEGF Family Ligand-Receptor Interactions	0,00	0,04
ErbB4 Signaling	0,00	0,03
Netrin Signaling	0,00	0,02
Triacylglycerol Degradation	0,00	0,02
tRNA Charging	0,00	0,03
Stearate Biosynthesis I (Animals)	0,00	0,02
Superpathway of Methionine Degradation	0,00	0,03
Gαi Signaling	0,00	0,03
Gαq Signaling	0,00	0,03
Regulation of Cellular Mechanics by Calpain Protease	0,00	0,03
Remodeling of Epithelial Adherens Junctions	0,00	0,01
Role of p14/p19ARF in Tumor Suppression	0,00	0,03
HIPPO signaling	0,00	0,04
Estrogen Receptor Signaling	0,00	0,04
ERK/MAPK Signaling	0,00	0,04
JAK/Stat Signaling	0,00	0,04
Phototransduction Pathway	0,00	0,02
Chemokine Signaling	0,00	0,04
Integrin Signaling	0,00	0,03
Glutamate Receptor Signaling	0,00	0,02
TGF-β Signaling	0,00	0,02
IL-6 Signaling	0,00	0,03
BMP signaling pathway	0,00	0,01
PD-1. PD-L1 cancer immunotherapy pathway	0,00	0,01
Cancer Drug Resistance By Drug Efflux	0,00	0,02
Th1 and Th2 Activation Pathway	0,00	0,03
Th1 Pathway	0,00	0,02
Th2 Pathway	0,00	0,03
Sirtuin Signaling Pathway	0,00	0,03
SPINK1 Pancreatic Cancer Pathway	0,00	0,03
NER Pathway	0,00	0,01
Apelin Pancreas Signaling Pathway	0,00	0,02
BAG2 Signaling Pathway	0,00	0,02
FAT10 Cancer Signaling Pathway	0,00	0,02
T Cell Exhaustion Signaling Pathway	0,00	0,02
Systemic Lupus Erythematosus In T Cell Signaling Pathway	0,00	0,02
Senescence Pathway	0,00	0,03
Inhibition of ARE-Mediated mRNA Degradation Pathway	0,00	0,02
GM-CSF Signaling	0,00	0,03
Ephrin Receptor Signaling	0,00	0,03

Lactate Dehydrogenase (LDH) release assay

Cytotoxicity of smoke-exposed cells was assessed on day 28, based on the measurement of lactate dehydrogenase (LDH) activity released from the cytosol of damaged cells into the supernatant. LDH release was analyzed using the Cytotoxicity Detection Kit (cat #11644793001 Roche, Indianapolis, USA) according to manufacturer instructions. Optical density (OD) was measured with a spectrophotometer at 490 nm (Spectramax M5e; Molecular Devices, San José, USA).

Figure S4

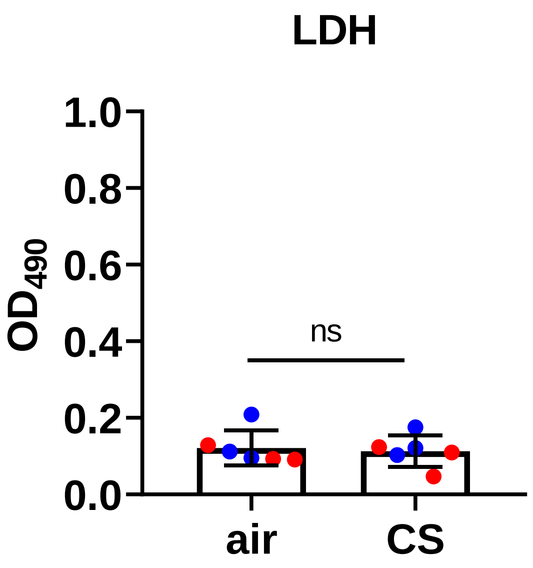

Lactate Dehydrogenase (LDH) release in CS- and air-exposed ALI cultures. OD_{490} measurements indicate LDH release in cell culture supernatants on day 28 after air-lift. Blue dots represent cultures from healthy individuals (n=3) and red dots represent cultures from COPD patients.

Figure S5

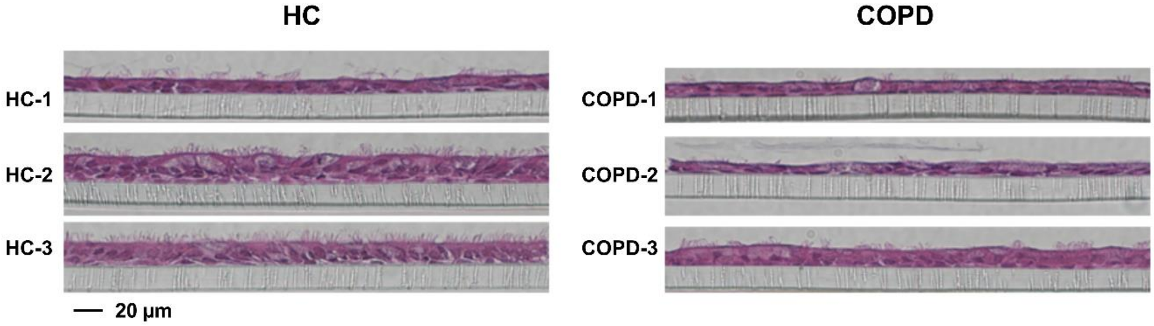

Hematoxylin & Eosin staining of SAEC ALI cultures from healthy donors (HC, n=3) and COPD patients (n=3). Cells were differentiated under ALI conditions for 28 days.

Figure S6

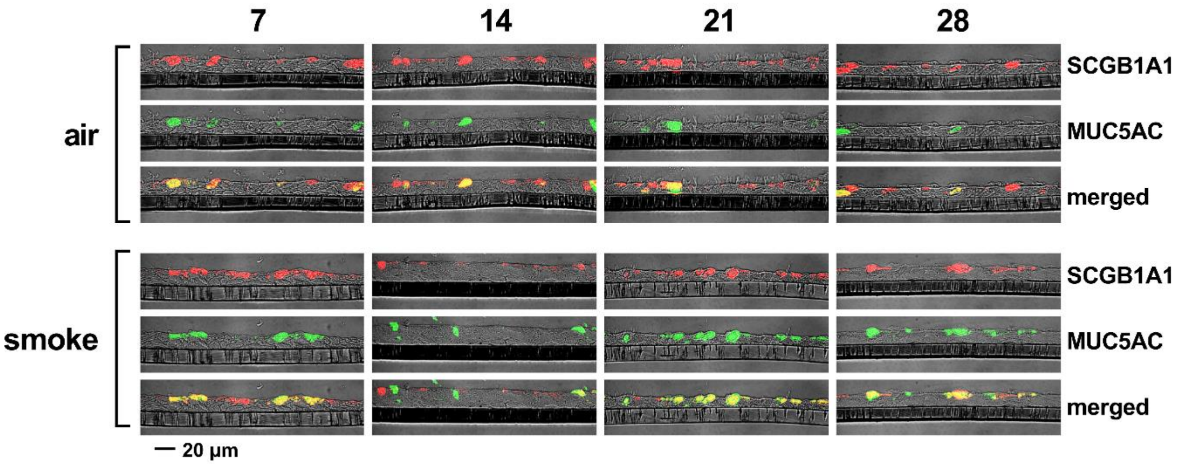

Immunofluorescent stainings of SAEC ALI cultures. Cells were intermittently exposed to smoke and air, respectively. Representative images of one healthy donor stained for SCGB1A1 (red) and MUC5AC (green) on day 7, 14, 21 and 28.

Table S4: RNA sequencing expression raw data. Reads per kilo base per million (RPKM) mapped reads of specific genes. Donors A, B and C are healthy controls; Donors D, E and F are COPD patients.

GeneID	GeneName	A_CS_1	A_CS_2	A_CS_3	A_air_1	A_air_2	A_air_3
ENSG00000013297	*CLDN11*	0,1	0,179	0,471	0,585	1,33	0,78
ENSG00000026508	*CD44*	78,417	93,959	103,296	61,997	54,429	55,892
ENSG00000066405	*CLDN18*	1,449	0,481	1,022	2,169	2,443	1,982
ENSG00000073282	*TP63*	18,583	24,627	22,859	23,864	24,136	21,594
ENSG00000122735	*DNAI1*	53,531	47,938	67,92	77,331	88,665	80,837
ENSG00000129654	*FOXJ1*	180,114	279,427	248,849	253,06	262,111	301,227
ENSG00000134873	*CLDN10*	130,776	119,742	90,868	22,286	23,061	23,079
ENSG00000149021	*SCGB1A1*	4937,77	7616,98	9798,84	18120,6	18099,1	17792,9
ENSG00000161055	*SCGB3A1*	1380,72	2819,5	2837,54	7300,1	6783,72	6317
ENSG00000163207	*IVL*	0,968	0,201	0,46	0,26	0,285	0,245
ENSG00000166086	*JAM3*	0,994	1,04	0,86	1,92	1,759	1,708
ENSG00000171401	*KRT13*	48,022	32,714	27,929	4,85	4,29	4,435
ENSG00000175793	*SFN*	110,66	80,472	62,463	32,125	36,519	31,356
ENSG00000181458	*TMEM45A*	75,635	76,526	101,41	105,662	139,898	138,834
ENSG00000181885	*CLDN7*	280,623	223,618	190,915	169,997	173,639	165,412
ENSG00000185499	*MUC1*	934,919	1157,05	1004,56	613,795	662,972	632,618
ENSG00000186081	*KRT5*	652,247	1043,56	776,853	439,411	382,089	352,922
ENSG00000186847	*KRT14*	3,286	2,866	1,84	0,283	0,117	0,137
ENSG00000197446	*CYP2F1*	41,644	78,646	99,199	201,36	219	215,447
ENSG00000205420	*KRT6A*	53,444	73,843	63,95	19,461	18,745	15,379
ENSG00000215182	*MUC5AC*	47,333	119,974	128,157	47,793	56,31	52,171

GeneID	GeneName	B_CS_1	B_CS_2	B_CS_3	B_air_1	B_air_2	B_air_3
ENSG00000013297	*CLDN11*	0,077	0,13	0,11	0,557	0,306	0,407
ENSG00000026508	*CD44*	73,956	117,293	105,629	47,865	46,442	41,458
ENSG00000066405	*CLDN18*	0,746	1,114	0,522	1,149	4,376	0,56
ENSG00000073282	*TP63*	21,403	23,892	24,352	24,989	24,684	25,617
ENSG00000122735	*DNAI1*	45,944	66,349	59,877	62,725	105,407	49,242
ENSG00000129654	*FOXJ1*	265,717	257,803	279,483	268,056	277,216	300,74
ENSG00000134873	*CLDN10*	162,359	119,177	99,145	17,311	15,377	15,926
ENSG00000149021	*SCGB1A1*	3247,7	5669,24	7256,57	19621,6	18995,3	23949,4
ENSG00000161055	*SCGB3A1*	1177,76	1781,08	3208,28	8385,71	7908,54	10660,7
ENSG00000163207	*IVL*	0,335	0,216	0,098	0,197	0,134	0,096
ENSG00000166086	*JAM3*	0,387	0,625	0,695	1,573	1,45	1,601
ENSG00000171401	*KRT13*	74,881	50,578	66,908	4,542	4,626	3,883
ENSG00000175793	*SFN*	127,023	80,682	91,903	33,635	28,086	30,478
ENSG00000181458	*TMEM45A*	17,639	20,324	22,589	48,134	49,094	53,806
ENSG00000181885	*CLDN7*	329,075	210,047	227,46	189,047	175,311	172,371
ENSG00000185499	*MUC1*	985,461	879,159	971,688	474,859	447,988	434,019
ENSG00000186081	*KRT5*	1194,01	1590,61	1757,38	572,443	442,707	513,33
ENSG00000186847	*KRT14*	5,992	2,548	2,443	0,117	0,193	0,214
ENSG00000197446	*CYP2F1*	24,569	52,347	59,596	217,748	242,12	281,455
ENSG00000205420	*KRT6A*	105,683	94,887	103,565	22,535	15,489	18,13
ENSG00000215182	*MUC5AC*	244,972	465,214	608,863	154,216	137,202	185,147

GeneID	GeneName	C_CS_1	C_CS_2	C_CS_3	C_air_1	C_air_2	C_air_3
ENSG00000013297	*CLDN11*	0,311	0,993	0,341	1,741	1,374	2,172
ENSG00000026508	*CD44*	56,871	63,754	145,492	32,115	37,925	28,812
ENSG00000066405	*CLDN18*	2,269	1,647	0,425	0,708	1,233	3,315
ENSG00000073282	*TP63*	14,629	18,263	33,591	14,864	15,403	12,607
ENSG00000122735	*DNAI1*	96,863	98,792	60,01	66,782	79,022	129,021
ENSG00000129654	*FOXJ1*	315,25	350,784	252,448	332,381	313,295	340,247
ENSG00000134873	*CLDN10*	103,532	57,186	78,737	10,313	11,254	10,025
ENSG00000149021	*SCGB1A1*	6172,71	7826,02	9526,48	15331	14316,8	14375,3
ENSG00000161055	*SCGB3A1*	3994,63	6563,41	5276,15	8994,12	8348,22	10493,2
ENSG00000163207	*IVL*	0,428	0,219	0,329	0,146	0,227	0,196
ENSG00000166086	*JAM3*	0,375	0,709	0,33	1,009	1,76	1,6
ENSG00000171401	*KRT13*	19,459	17,314	48,781	4,984	5,679	6,089
ENSG00000175793	*SFN*	70,035	65,606	135,712	27,759	29,062	26,265
ENSG00000181458	*TMEM45A*	77,563	59,296	101,688	100,506	100,288	80,572
ENSG00000181885	*CLDN7*	278,808	274,367	267,066	191,526	202,879	207,665
ENSG00000185499	*MUC1*	1150,12	1103,55	1179,79	576,687	603,982	581,514
ENSG00000186081	*KRT5*	435,212	733,9	1346,5	232,691	259,967	216,444
ENSG00000186847	*KRT14*	0,48	0,751	2,065	0,041	0,091	0
ENSG00000197446	*CYP2F1*	48,196	65,788	75,509	148,511	149,544	148,347
ENSG00000205420	*KRT6A*	30,74	42,287	113,208	11,447	13,882	9,522
ENSG00000215182	*MUC5AC*	85,875	181,1	207,506	37,325	44,329	46,598

GeneID	GeneName	D_CS_1	D_CS_2	D_CS_3	D_air_1	D_air_2	D_air_3
ENSG00000013297	*CLDN11*	0,267	0,064	0,2	2,593	2,524	5,203
ENSG00000026508	*CD44*	82,687	109,338	94,929	63,134	60,714	72,346
ENSG00000066405	*CLDN18*	0,172	0,124	0,171	0,953	0,968	0,558
ENSG00000073282	*TP63*	40,874	39,608	37,594	44,277	42,155	44,236
ENSG00000122735	*DNAI1*	20,901	35,273	39,876	45,555	49,88	39,251
ENSG00000129654	*FOXJ1*	147,221	233,938	228,201	219,906	251,987	260,996
ENSG00000134873	*CLDN10*	124,088	135,772	73,049	17,398	16,272	17,052
ENSG00000149021	*SCGB1A1*	6709,46	12110,9	15012,2	33465,6	31154,6	33597,6
ENSG00000161055	*SCGB3A1*	3019,51	4991,38	8699,69	12167,9	13102,8	17325,1
ENSG00000163207	*IVL*	0,717	0,16	0,199	0,1	0,244	0,17
ENSG00000166086	*JAM3*	0,312	0,317	0,638	1,235	1,242	0,873
ENSG00000171401	*KRT13*	156,513	44,914	69,038	11,155	11,12	15,16
ENSG00000175793	*SFN*	203,352	86,419	106,022	46,249	47,647	53,99
ENSG00000181458	*TMEM45A*	51,176	66,971	58,11	117,258	122,613	113,964
ENSG00000181885	*CLDN7*	369,033	233,797	245,941	188,933	189,248	196,17
ENSG00000185499	*MUC1*	1345,08	1504,53	1395,09	645,117	663,239	690,175
ENSG00000186081	*KRT5*	1471,3	1605,62	1800,81	680,228	637,973	766,243
ENSG00000186847	*KRT14*	7,38	0,993	1,636	0,108	0,166	0,251
ENSG00000197446	*CYP2F1*	32,992	76,144	85,824	304,773	299,998	337,144
ENSG00000205420	*KRT6A*	82,954	66,919	69,703	22,947	18,979	25,037
ENSG00000215182	*MUC5AC*	131,25	289,051	310,727	82,443	84,088	95,704

GeneID	GeneName	E_CS_1	E_CS_2	E_CS_3	E_air_1	E_air_2	E_air_3

ENSG00000013297	*CLDN11*	0,405	0,085	1,04	2,326	1,554	2,508
ENSG00000026508	*CD44*	288,389	213,773	257,546	158,95	117,151	154,856
ENSG00000066405	*CLDN18*	0,235	1,553	2,947	4,232	6,329	6,276
ENSG00000073282	*TP63*	43,255	34,757	27,566	23,913	21,838	25,268
ENSG00000122735	*DNAI1*	4,074	53,63	67,123	103,511	130,937	128,151
ENSG00000129654	*FOXJ1*	7,529	138,188	59,408	143,126	169,764	146,914
ENSG00000134873	*CLDN10*	84,907	262,151	200,015	42,224	40,99	38,093
ENSG00000149021	*SCGB1A1*	2195,49	6319,56	5902,81	14068,8	15942,9	12779,1
ENSG00000161055	*SCGB3A1*	117,837	924,293	466,847	2167,88	3250,95	2197,35
ENSG00000163207	*IVL*	4,38	0,632	1,111	1,149	0,399	1,506
ENSG00000166086	*JAM3*	0,364	0,634	0,729	1,424	1,258	1,593
ENSG00000171401	*KRT13*	12,801	54,332	3,25	37,949	36,676	34,879
ENSG00000175793	*SFN*	277,687	177,518	124,591	76,522	60,411	74,494
ENSG00000181458	*TMEM45A*	66,999	111,862	155,658	171,067	125,049	136,264
ENSG00000181885	*CLDN7*	345,084	272,939	216,653	206,285	193,751	202,448
ENSG00000185499	*MUC1*	488,677	1121,16	850,894	769,923	723,345	724,505
ENSG00000186081	*KRT5*	1003,6	1311,9	597,805	632,051	601,5	608,341
ENSG00000186847	*KRT14*	2,77	5,216	0,543	0,104	0,185	0,381
ENSG00000197446	*CYP2F1*	0,807	54,489	33,124	164,827	207,422	157,519
ENSG00000205420	*KRT6A*	169,377	251,469	112,003	40,32	47,885	43,144
ENSG00000215182	*MUC5AC*	77,905	1340,1	833,531	224,477	289,928	250,647

GeneID	GeneName	F_CS_1	F_CS_2	F_CS_3	F_air_1	F_air_2	F_air_3
ENSG00000013297	*CLDN11*	0,358	0,287	0,384	0,669	0,989	1,088
ENSG00000026508	*CD44*	65,419	74,553	80,935	64,436	57,335	87,046
ENSG00000066405	*CLDN18*	0,676	1,05	2,128	1,364	2,069	1,192
ENSG00000073282	*TP63*	31,765	27,696	27,177	37,206	42,134	35,378
ENSG00000122735	*DNAI1*	43,311	69,23	94,968	61,07	77,784	65,834
ENSG00000129654	*FOXJ1*	255,459	219,722	236,517	201,643	206,877	273,567
ENSG00000134873	*CLDN10*	70,597	41,287	33,505	11,326	9,009	13,037
ENSG00000149021	*SCGB1A1*	5592,49	13448,5	13833,8	23705,6	24258,1	21949,6
ENSG00000161055	*SCGB3A1*	2095,86	4180,31	5470,54	9081,79	11025,5	10059,5
ENSG00000163207	*IVL*	0,134	0,081	0,101	0,113	0,129	0,286
ENSG00000166086	*JAM3*	1,374	2,247	2,417	3,023	4,406	4,392
ENSG00000171401	*KRT13*	39,035	10,966	12,204	6,334	4,454	8,083
ENSG00000175793	*SFN*	112,143	47,547	44,403	36,813	33,006	44,799
ENSG00000181458	*TMEM45A*	39,018	79,072	65,844	123,525	106,223	122,69
ENSG00000181885	*CLDN7*	273,858	198,627	197,939	165,371	155,57	206,079
ENSG00000185499	*MUC1*	1027,46	904,383	717,171	564,355	498,851	594,853
ENSG00000186081	*KRT5*	1055,27	724,231	709,018	637,164	596,073	651,141
ENSG00000186847	*KRT14*	1,693	0,498	0,427	0,085	0,109	0,133
ENSG00000197446	*CYP2F1*	46,09	119,09	148,36	258,134	301,633	292,871
ENSG00000205420	*KRT6A*	39,26	17,508	20,644	10,021	9,081	22,458
ENSG00000215182	*MUC5AC*	46,522	64,57	61,007	10,93	8,332	23,218

References

1 Smith, C. M. *et al.* ciliaFA: a research tool for automated, high-throughput measurement of ciliary beat frequency using freely available software. *Cilia* **1**, 14, doi:10.1186/2046-2530-1-14 (2012).

2 Meste, O., Brau, F. & Guyon, A. Robust estimation of the motile cilia beating frequency. *Medical & biological engineering & computing* **53**, 1025-1035, doi:10.1007/s11517-015-1345-0 (2015).

3 Jing, J. C., Chen, J. J., Chou, L., Wong, B. J. F. & Chen, Z. Visualization and Detection of Ciliary Beating Pattern and Frequency in the Upper Airway using Phase Resolved Doppler Optical Coherence Tomography. *Scientific reports* **7**, 8522, doi:10.1038/s41598-017-08968-x (2017).

4 Norina, S. B., Ageev, V. G. & Rastopov, S. F. *Motility and ciliary beating frequency detection of cells and invertebrates for environmental biomonitoring*. Vol. 3196 EBO (SPIE, 1998).
